# Gestational hypertension increases risk of seizures in children and mice

**DOI:** 10.1172/JCI183393

**Published:** 2025-06-16

**Authors:** Baojian Xue, Serena B. Gumusoglu, Grant Tiarks, Brittany P. Todd, Angela Wong, Donna A. Santillan, Chin-Chi Kuo, Hsiu-Yin Chiang, Rohith Ravindranath, Sophia Y. Wang, Vinit B. Mahajan, Alan Kim Johnson, Heath A. Davis, Polly Ferguson, Elizabeth A. Newell, Mark K. Santillan, Jason M. Misurac, Alexander G. Bassuk

**Affiliations:** 1 Stead Family Department of Pediatrics,; 2 Department of Obstetrics and Gynecology,; 3 Department of Psychiatry, and; 4 The Hawk Intellectual and Developmental Disabilities Research Center, Roy J. and Lucille A. Carver College of Medicine, University of Iowa, Iowa City, Iowa, USA.; 5 Big Data Center,; 6 Department of Biomedical Informatics, and; 7 Department of Internal Medicine, China Medical University Hospital, Taichung, Taiwan.; 8 Byers Eye Institute, and; 9 Molecular Surgery Laboratory, Stanford University, Palo Alto, California, USA.; 10 Department of Psychological and Brain Sciences,; 11 Institute for Clinical and Translational Science-Biomedical Informatics,; 12 The Iowa Neuroscience Institute, and; 13 The Department of Neurology, Roy J. and Lucille A. Carver College of Medicine, University of Iowa, Iowa City, Iowa, USA.

**Keywords:** Inflammation, Neuroscience, Hypertension, Seizures

## Abstract

Gestational hypertension (GH) is prevalent, with life-long health burdens for mothers and their children exposed in utero. We analyzed the nation-wide Epic Cosmos dataset and found significantly higher rates of seizures in children of mothers with GH than in children of normotensive mothers. Complementary studies of nested Iowa and Stanford cohorts and a large Taiwanese cohort also revealed significantly increased seizure risk after covariate adjustments. We modeled this association in an angiotensin (ANG) II mouse model of GH. Maternal ANG II significantly increased seizure grade and deaths elicited by pilocarpine among male but not female offspring. Electrical stimulation increased seizure grade and death across sexes in offspring from ANG II–treated dams. Proinflammatory and microglial gene expression in the brain were upregulated only in male offspring from ANG II–treated dams. Chronic phenylephrine, a GH model lacking the maternal proinflammatory aspects of ANG II, induced similar offspring seizure phenotypes. PLX5622-induced depletion of microglia or antiinflammatory pentoxifylline abolished this sensitized seizure response and lowered mortality in the ANG II model. These results suggest that GH programs offspring risk for seizures in a sex-dependent manner in humans and mice. Neuroinflammatory mechanisms may contribute to the elevated sensitivity and mortality from seizures elicited by GH exposure in utero.

## Abstract 2

This study indicates that maternal gestational hypertension increases the risk for seizures in both children and mice.

## Graphical abstract

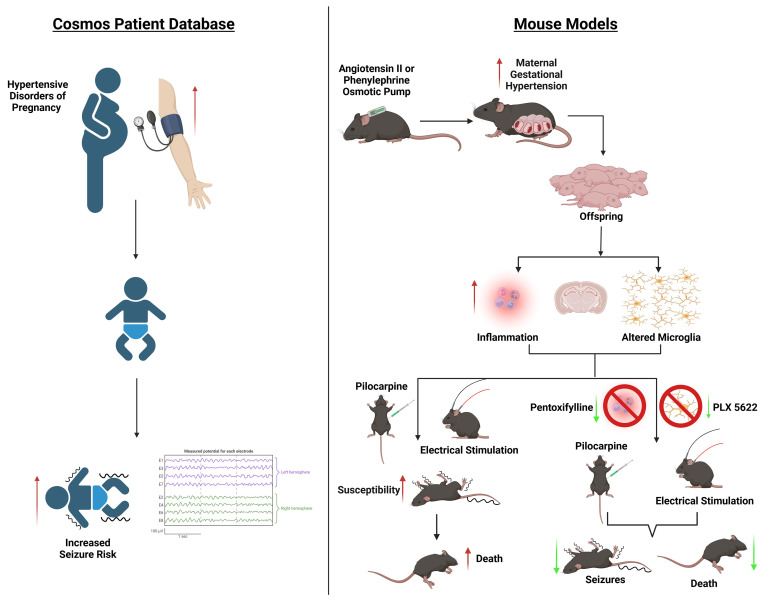

## Introduction

Hypertensive disorders of pregnancy (HDP), including severe forms such as preeclampsia, are common conditions affecting nearly 16% of deliveries at US hospitals (during 2017–2019) and even more among minoritized populations (1). Exposure to these disorders in fetal life increased the risk for neurodevelopmental, psychiatric, and neurologic sequelae in some offspring (2). Human cohort studies reveal an increased risk for seizures in children who are prenatally exposed to preeclampsia (3–6), and this risk correlates positively with the severity of the preeclampsia (5–7). Moreover, more than 2% of all children have seizures (8–10), the causes of which are poorly understood. These findings raise the question of whether gestational hypertension (GH) of any variety during pregnancy is an important risk factor for childhood seizures and, furthermore, whether this association may reveal underlying candidate mechanisms. However, there is a paucity of clinical data on the relationship between GH and seizure risk among exposed children and few validated animal models to investigate the causal effects of GH on offspring seizure pathobiology and the related CNS mechanisms triggered by GH.

Epilepsy is a chronic neurological disorder characterized by recurrent seizures. Previous studies of epilepsy have focused mainly on an imbalance between neuronal excitation and inhibition (11, 12). However, microglia, resident immune cells of the central nervous system (CNS) undergo significant changes in state and density in the epileptic brain (13), and neuroinflammation has been implicated in seizures and epileptogenesis (14). Studies analyzing the brains of seizing patients and animal models of seizures and epilepsy reveal increased microglia numbers and activation, leading to increased production of inflammatory cytokines within 4 hours of seizure onset, especially in the hippocampus (15–17). One recent study further demonstrated that proinflammatory mechanisms contribute to the pathogenesis of refractory epilepsy (nonresponsive to antiepileptic drugs) (18). Complementary work finds that pharmacogenetic inhibition of microglial recruitment and activation, or administration of antiinflammatory medication, reduces seizure severity (19–22). These results suggest that neuroinflammation is involved in the pathogenesis and progression of seizure disorders. However, whether microglial activation and concomitant cytokine release represents a primary cause versus a secondary, reinforcing mechanism in seizures is not clear. Furthermore, the question of an intergenerational link, that is, whether GH-mediated neuroimmune programming occurs in the fetus and increases seizure risk postnatally remained unanswered.

In this study, we first investigated if there was an association between prenatal exposure to a hypertensive maternal environment and the incidence of pediatric seizures utilizing 4 complementary cohorts. Together, these cohorts contain nearly 250 million patient records. First, in the Epic Cosmos electronic health record dataset (>246 million patients across >1,415 hospitals in the US and Lebanon), we identified a highly significant (*P* < 0.001) association between GH and seizure incidence in children. Second, we validated this association in 2 smaller, but richly annotated, US-based cohorts, namely, the Iowa Intergenerational Health Knowledgebase (IGHK) and a Stanford-based healthcare system cohort (extracted from STARR-OMOP, the Stanford Electronic Health Records research database). For these 2 smaller cohorts, the maternal-child data are linked, allowing for multivariate regression. Both cohorts are nested within the broader Cosmos dataset but offer more granular and controlled analyses than are available in Cosmos. Finally, to determine whether the association between GH and pediatric seizure replicates beyond the United States, we tested this association in Taiwan’s National Health Insurance Database, which contains over 2 million linked maternal-child dyads from 2000–2015. Here, we also found a robust and significant association between GH and childhood seizure, even after adjusting for relevant covariates. In the Cosmos cohort, male offspring were over-represented relative to female offspring among seizure groups (*P* < 0.001), irrespective of maternal hypertension.

Finally, to provide insights into causal mechanisms and therapeutics, we explored this clinical connection using 2 complementary mouse models of GH. The first model, angiotensin (ANG) II infusion during pregnancy, induced hypertension and systemic proinflammation in dams (23) and provided an entry point to test whether fetal programming of seizure risk might be mediated by neuroimmune mechanisms. In this ANG II mouse model, GH increased offspring sensitivity and mortality to chemically and electrically induced seizures. The severity of these effects depended, at least in part, on increased microglial numbers and inflammation within the hippocampus. Either depletion of microglia or inhibition of inflammation more broadly ameliorated offspring seizure phenotypes. Our study also revealed significant sex differences in the incidence, sensitivity, and mortality due to seizures induced by prenatal exposure to ANG II maternal hypertension.

We complemented the ANG II model with another hypertension model that has more acute effects, a distinct mechanism of action, and less inflammatory involvement in dams: chronic maternal phenylephrine (PE). ANG II is a peptide hormone which induces hypertension both directly (via vasoconstriction) and indirectly (via the effects of aldosterone secretion on sodium and water retention and via proinflammatory cascades) (23). In contrast to ANG II, PE is an α-1 adrenergic receptor agonist that causes hypertension by binding blood vessel receptors to cause vasoconstriction and increased vascular resistance (24). Critically, PE does not induce many of the nonphysiologic effects of ANG II infusion in dams, such as cerebrovascular dysfunction, endoplasmic reticulum, oxidative stress, and significant systemic proinflammation (25–27). As with the ANG II model, PE-induced GH increased neuroinflammatory and microglial gene expression in offspring and lowered offspring seizure thresholds. However, in contrast to ANG II offspring, the PE offspring did not appear to exhibit changes to the renin-angiotensin system (RAS) in the brain. These results suggest that GH, per se, and not maternal hypertension with additional systemic aspects (e.g., the nonphysiologic effects of ANG II), is sufficient to increase seizure severity and mortality in prenatally exposed offspring.

Together, this work elucidates a relationship between GH and the risk of seizures in offspring. By leveraging extensive human cohort data and complementary animal models, we uncovered a potential neuroimmune mechanism that may mediate this association. Our findings not only enhance the understanding of the pathophysiology of pediatric seizure but also inform the development of targeted therapeutic interventions to mitigate seizure risk in children exposed to hypertension in utero.

## Results

### Increased seizures among children from hypertensive pregnancies in 4 large clinical datasets.

To first identify whether there is an overall association between maternal hypertension exposure and childhood seizure, we used the Epic Cosmos database of 246 million individuals treated across 1,400 hospitals and 33,000 clinics in the United States and Lebanon. We identified 229,357 individuals (below 18 years of age) with a diagnosis of seizures among 7,257,078 children with birth parent data available. The rate of seizure was 50,288 of 1,365,254 (3.68%) in patients born to mothers who were hypertensive during pregnancy and 179,069 of 5,891,824 (3.04%) in patients born to mothers without hypertension during pregnancy. The χ^2^ contingency test indicated that exposure to maternal hypertension was significantly associated with a higher incidence of seizures (OR 1.22; 95% CI: 1.21–1.23, *P* < 0.001) in children. Moreover, male offspring were overrepresented relative to female offspring among the seizure groups (*P* < 0.001), regardless of maternal hypertension.

While these analyses did not produce line-level data allowing for covariate adjustment, we found that the rate of obesity was higher in mothers with hypertension than in those without (43.0% vs 16.3%; *P* < 0.001), as was maternal diabetes (20.4% vs 8.4%; *P* < 0.001). We also found that the rate of developmental delay was higher in children from a hypertensive pregnancy than in those from normotensive pregnancies (10.3% vs. 7.7%; *P* < 0.001), an association observed in children either with (31.3% vs 25.8%; *P* < 0.001) or without (9.5% vs 7.1%; *P* < 0.001) a seizure diagnosis. Full demographic information and statistical comparisons are presented in Table 1 and Supplemental Tables 1–4 (supplemental material available online with this article; https://doi.org/10.1172/JCI183393DS1).

To determine whether the association between GH and child seizure risk survived covariate adjustment, we next examined this association within a richly annotated clinical knowledgebase — the IGHK. In this database, pregnant women whose neonates experienced seizures (*n* = 1,370) had significantly higher rates of maternal body mass index (BMI) (16% vs. 12%; *P* < 0.001), adverse neonatal outcomes (59% vs. 35%; *P* < 0.001), and HDP (37% vs. 33%; *P* < 0.001) compared with controls (*n* = 34,297). After controlling for age, gravida, BMI, diabetes, and ancestry, pregnant women affected by HDP had higher odds of having a child with seizures than did pregnant women without HDP (adjusted OR = 1.132 [1.003–1.278], *P* < 0.05) (Table 2 and Supplemental Tables 5 and 6).

We next expanded our study to another large, US-based clinical data repository: the Stanford Research Data Repository Electronic Health Records Observational Medical Outcomes Partnership (STARR-OMOP). The STARR-OMOP is the Stanford Electronic Health Records research database consisting of linked maternal-child data from both the adult Stanford Healthcare system and the Lucile Packard Children’s Hospital system. In this dataset, maternal hypertension significantly predicted a child’s (<18 years of age) risk of seizures, even after adjusting for maternal age, maternal BMI above 40, diabetes in pregnancy, and ancestry (adjusted OR 1.36; 95% CI: 1.21–1.52, *P* < 0.001). Full cohort characteristics and statistics are presented in Table 3 and Supplemental Tables 7 and 8.

Finally, to test whether maternal hypertension is associated with child seizures across diverse settings, we examined this association in a large international cohort: Taiwan’s National Health Insurance Database of 2,003,354 mother-child pairs. After adjusting for maternal age, maternal diabetes, maternal obesity, gestational age at delivery, infant birth weight, infant 5-minute Apgar score, and childhood development delay, we found that maternal hypertension was significantly associated with an increased risk of childhood seizure (adjusted OR 1.17; 95% CI: 1.14–1.20, *P* < 0.001). Full cohort characteristics and statistics are presented in Table 4 and Supplemental Tables 9–11.

Together, our studies across 4 complementary cohorts (Epic Cosmos, IGHK, Stanford STARR-OMOP, and Taiwan’s National Health Insurance Database) link maternal hypertension exposure to pediatric seizure occurrence in the next generation.

### ANG II or PE is sufficient to cause hypertension in murine pregnancy.

**Figure 1:**
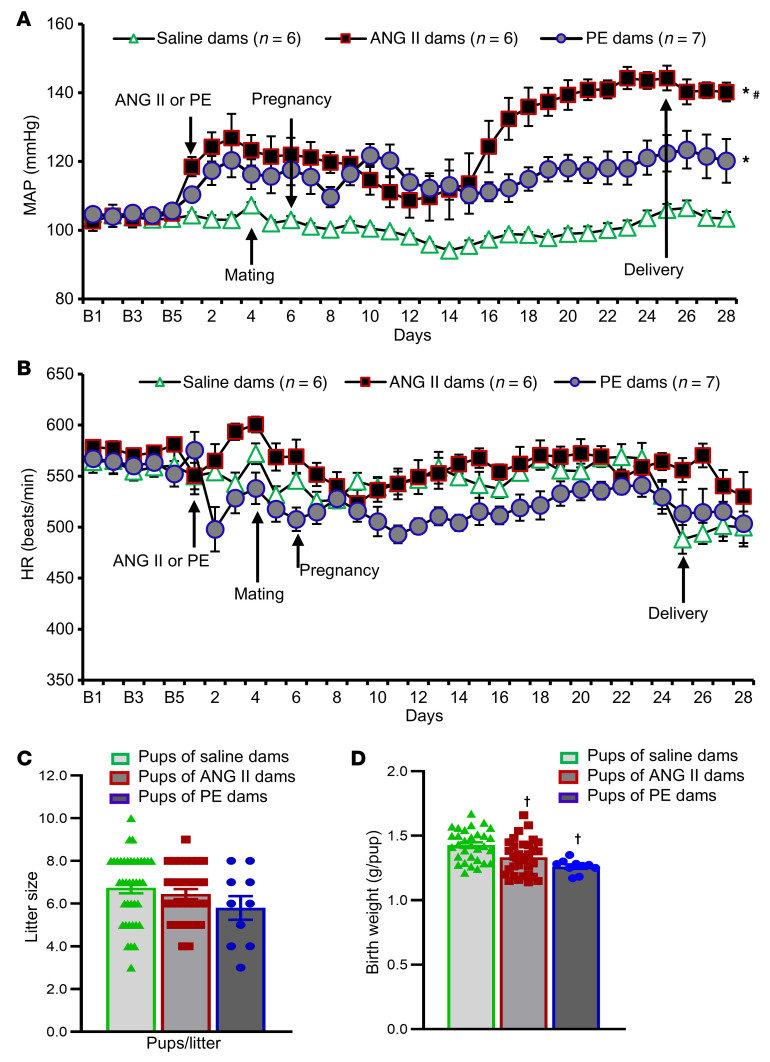
Maternal ANG II– or PE-induced GH and their effects on the neonates. Changes in MAP (**A**) and HR (**B**) in mouse dams with chronic infusion of saline, ANG II, or PE during pregnancy. (**C** and **D**) Litter size and birth weights. Two-way ANOVA; *P < 0.05 vs. saline dams; ^#^*P* < 0.05 vs. PE-treated dams; ^†^*P* < 0.05 vs. pups of saline-treated dams. Data are shown as the mean ± SEM.

To complement our clinical analyses, we next utilized 2 complementary animal GH models, the chronic ANG II model and the PE model. Both models have been validated to induce hypertension in mice. There were no differences in baseline blood pressure (BP) or heart rate (HR) between saline- and ANG II–treated dams. During pregnancy, systolic BP, diastolic BP, and mean arterial pressure (MAP) were significantly increased in ANG II-infused females compared with saline-infused control females (*P* < 0.05, Figure 1A and Supplemental Figure 1), but there were no differences in HR or locomotor activity (Figure 1B and Supplemental Figure 1). ANG II–induced GH did not impair reproduction or skew offspring sex ratios. In total, 31 dams with ANG II–induced hypertension produced 200 pups including 102 males and 98 females, whereas 39 normotensive dams produced a total of 263 pups including 134 males and 129 females. There were no significant differences in litter size by condition (6.7 ± 0.3 pups vs. 6.5 ± 0.2 pups, Figure 1C). However, the average weight of ANG II pups on the day of birth (P0) was less than that of control pups (1.33 ± 0.03 g/pup vs. 1.43 ± 0.02 g/pup, respectively, *P* < 0.05, Figure 1D).

In the PE-induced GH model, 10 dams with PE-induced hypertension produced 58 pups including 31 males and 27 females. PE infusion in pregnancy significantly increased MAP and systolic BP, though not to the degree of that induced by ANG II (*P* < 0.05, Figure 1A and Supplemental Figure 1). We noted no differences in diastolic BP, HR, or locomotor activity in PE-infused females compared with control females (Figure 1B and Supplemental Figure 1). Similar to ANG II–treated dams, PE-treated dams had normal litter sizes (5.8 ± 0.3 pups/litter, Figure 1C) but lower P0 pup birth weights (1.26 ± 0.02 g/pup; *P* < 0.05, Figure 1D) relative to saline control–treated dams.

### Expression of proinflammatory and microglial genes in hippocampi of young and adult mouse offspring was altered by prenatal hypertension exposure.

**Figure 2:**
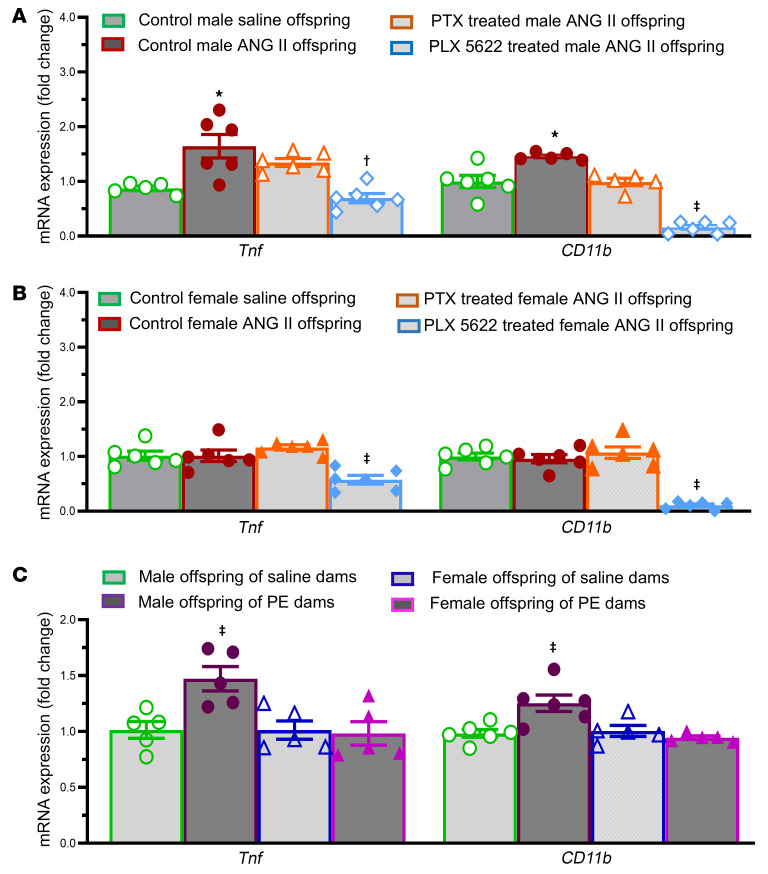
Hippocampal pro-inflammation with prenatal ANG II or PE and modulation by PTX or PLX5622. Quantitative comparison of the mRNA expression of proinflammatory cytokines (*Tnf*) and a microglial marker (*Cd11b*) in the hippocampus of adult offspring (10 weeks old) from normotensive (saline-treated) dams and from dams with ANG II–induced hypertension (male offspring, **A** and female offspring, **B**) or PE-induced hypertension (offspring of both sexes, **C**) (*n* = 5–6/group). One-way ANOVA; *P < 0.05 vs. male offspring of saline-treated dams; ^†^*P* < 0.05 vs. control ANG II offspring; ^‡^*P* < 0.05 vs. other 3 groups of offspring. Data are shown as the mean ± SEM.

To better understand the neurobiological changes that might drive neuropathology in hypertension-exposed offspring, we next assessed inflammatory gene expression in ANG II–exposed, PE-exposed, and control offspring brains. Hippocampi were dissected from the mice at 4 or 10 weeks of age. Male offspring from ANG II dams exhibited increased mRNA expression of proinflammatory (tumor necrosis factor [*Tnf*]) and microglial (cluster of differentiation molecule 11b/integrin α M [*Cd11b/Itgam*]) markers in the hippocampus when compared with saline controls (*P* < 0.05, Figure 2A and Supplemental Figure 2A). In contrast, ANG II–induced maternal hypertension had no effect on the expression of these genes in female offspring (*P* > 0.05, Figure 2B and Supplemental Figure 2A). Similarly, PE-induced maternal hypertension also upregulated expression of *Tnf* and *Cd11b* in hippocampi collected at 10 weeks of age in male but not female offspring (*P* < 0.05, Figure 2C).

We next evaluated the effect of pentoxifylline (PTX), a phosphodiesterase inhibitor that reduces the production of proinflammatory cytokines such as TNF in the periphery and the CNS (28–30). Two weeks of chronic PTX treatment (150 mg/kg/day in drinking water) had no effect on mRNA expression in the hippocampus of adult offspring from ANG II–treated dams (*P* > 0.05). In contrast, hippocampal expression of *Tnf* and *Cd11b* was significantly decreased in both male and female adult offspring of ANG II–treated dams (*P* < 0.05) after 1 week of treatment with the CSF 1 receptor (CSF1R) inhibitor PLX5622, which depletes microglia; in males, PLX5622 treatment restored hippocampal expression of *Tnf* to control levels (Figure 2, A and B).

### Expression of AT1-R in adult offspring hippocampus was differentially altered in PE and ANG II hypertension models.

To further differentiate the neurobiological changes in the hippocampi of ANG II–treated, PE-treated, and control offspring, we assessed the expression of 1 component of the RAS, the ANG II type 1 receptor (*At1r*). In hippocampi collected at 10 weeks of age, male but not female offspring of ANG II–treated dams had significantly upregulated *At1r* expression (*P* < 0.05, Supplemental Figure 3). In contrast, *At1r* expression in both male and female offspring of PE-treated dams was not altered. These results suggest that the maternal ANG II– and PE-induced hypertension models had different effects on the neurodevelopment and RAS programming of offspring.

### Sensitivity to seizure and attendant mortality in young offspring of GH pregnancies are increased.

Given the increased seizure risk in a large clinical cohort of maternal hypertension–exposed children and the increased neuroinflammation in our animal models of GH, we next examined seizure susceptibility in offspring from the ANG II model. Pilocarpine was used to induce seizures in young (4–5 weeks of age) female and male offspring, and the severity of seizures was scored on a modified Racine scale (31). There were no significant differences in Racine grades between male and female offspring from saline-treated dams following pilocarpine injections at any dose. However, both male and female offspring of ANG II–treated dams had Racine grade 3, 4, and 5 seizures following low cumulative doses (100–250 mg/kg) of pilocarpine. The Racine grades after a cumulative dose of 450–500 mg/kg of pilocarpine in male, but not female, offspring of ANG II–treated dams remained high when compared with same-sex offspring of saline-treated dams (*P* < 0.05, Supplemental Figure 2B). Pilocarpine injections did not cause death among these offspring.

We next used electrical stimulation (ES) as an independent method of seizure induction. Seizures and death were assessed in young offspring by applying current with stimulus intensities of 1–10 mA. Offspring from ANG II–treated dams showed a dose-dependent enhancement of Racine grades when compared with offspring from saline-treated dams (*P* < 0.05, Supplemental Figure 2C). Furthermore, offspring from ANG II–treated dams were more likely to die and died at a lower stimulus intensity (5 mA) than did the offspring of saline-treated dams (no deaths at 5 mA, some deaths at 10 mA; *P* < 0.05, Supplemental Figure 2D). There were no sex differences by either Racine grading or seizure-induced mortality, and this was true for offspring of both saline- and ANG II–treated dams.

### Increased sensitivity to seizure and attendant mortality in adult mouse offspring of GH pregnancies.

**Figure 3:**
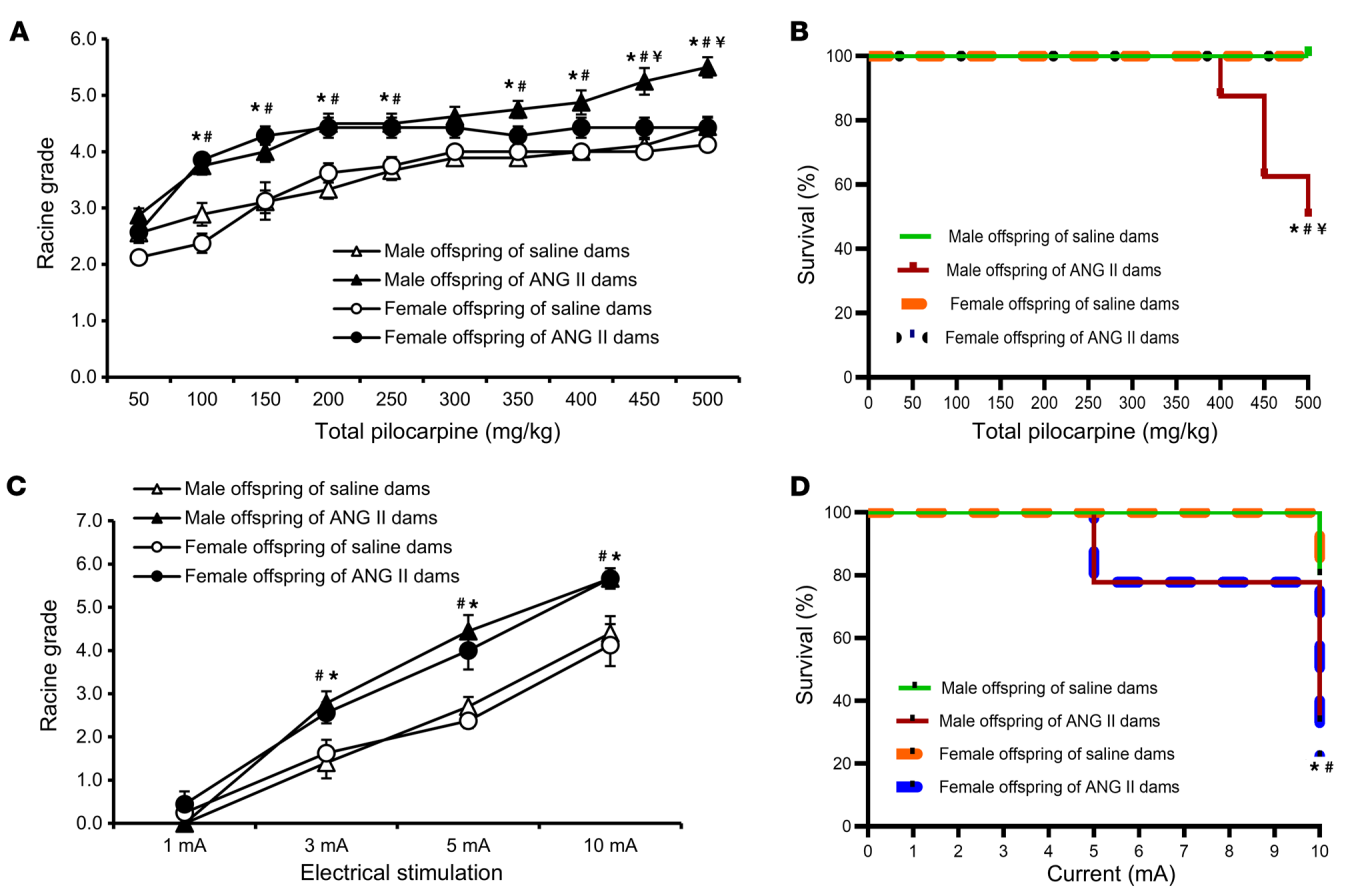
Seizure induction in offspring from ANG II–treated dams. Increased sensitivity and mortality to pilocarpine-induced (**A** and **B**) or ES-induced (**C** and **D**) seizures in adult male and female offspring of dams with ANG II–induced hypertension when compared with offspring of normotensive (saline-treated) dams. Sex differences in the sensitivity and mortality induced by pilocarpine were noted (*n* = 7–10/group). Two-way ANOVA followed by Tukey’s test; *P < 0.05 vs. male offspring of saline-treated dams; ^#^*P* < 0.05 vs. female offspring of saline-treated dams; ^¥^*P* < 0.05 vs. female offspring of ANG II–treated dams. Data are shown as the mean ± SEM.

To complement the studies of young offspring of the ANG II model and to determine the persistence of seizure phenotypes, we next tested seizure susceptibility of adult offspring from ANG II and PE models. Like the results in young female offspring of ANG II–treated dams, pilocarpine injections induced a similar pattern of elevated Racine grades with no deaths for adult female offspring (10–12 weeks of age) of ANG II–treated dams. However, a cumulative dose of 400–500 mg/kg of pilocarpine resulted not only in significantly enhanced Racine grades, but also a 50% death rate for the male offspring of ANG II–treated dams, which was sex specific (*P* < 0.05, Figure 3, A and B).

Interestingly, we noted no sex differences in Racine grades or mortality with respect to seizures induced by ES in either adult offspring of saline-treated dams or adult offspring of ANG II–treated dams. However, Racine grades and mortality in response to ES were higher in the offspring of ANG II–treated dams (*P* < 0.05, Figure 3, C and D) compared with the offspring of saline-treated dams.

To confirm that ES-associated death in offspring was from seizures, we treated offspring with the antiseizure drug diazepam 30 minutes before application of ES. Diazepam prevented ES-associated death in all groups of adult offspring (Supplemental Figure 4).

**Figure 4:**
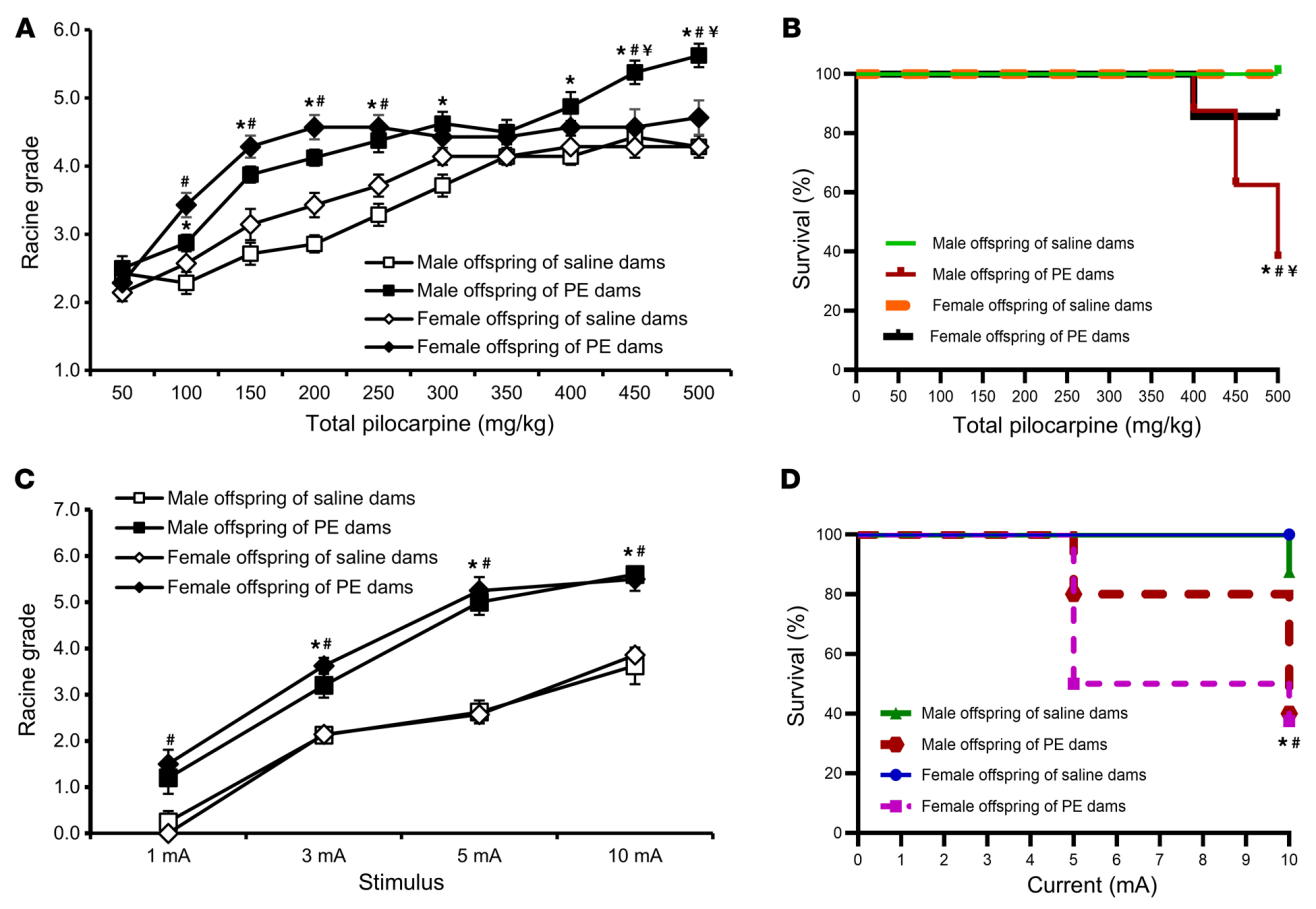
Seizure induction in offspring from PE–treated dams. Increased sensitivity and mortality to pilocarpine-induced (**A** and **B**) or ES-induced (**C** and **D**) seizures in adult male and female offspring of dams with PE–induced hypertension when compared with offspring of normotensive (saline-treated) dams. There were sex differences in the sensitivity and mortality induced by pilocarpine (*n* = 7–10/group). Two-way ANOVA followed by Tukey’s test; *P < 0.05 vs. male offspring of saline-treated dams; ^#^*P* < 0.05 vs. female offspring of saline-treated dams; ^¥^*P* < 0.05 vs. female offspring of PE-treated dams. Data are shown as the mean ± SEM.

To complement the ANG II model, we also performed seizure induction studies in the PE model of GH, which produces hypertension in dams with much lower systemic inflammatory effects in the dams (26). As with ANG II treatment, PE-induced GH elicited higher Racine grades and more deaths by pilocarpine administration for adult male versus female offspring (*P* < 0.05, Figure 4, A and B). ES also resulted in elevated Racine grades and higher mortality for both male and female offspring from the PE model (*P* < 0.05, Figure 4, C and D).

### Antiinflammatory drug attenuates seizures in adult offspring of GH pregnancies.

**Figure 5:**
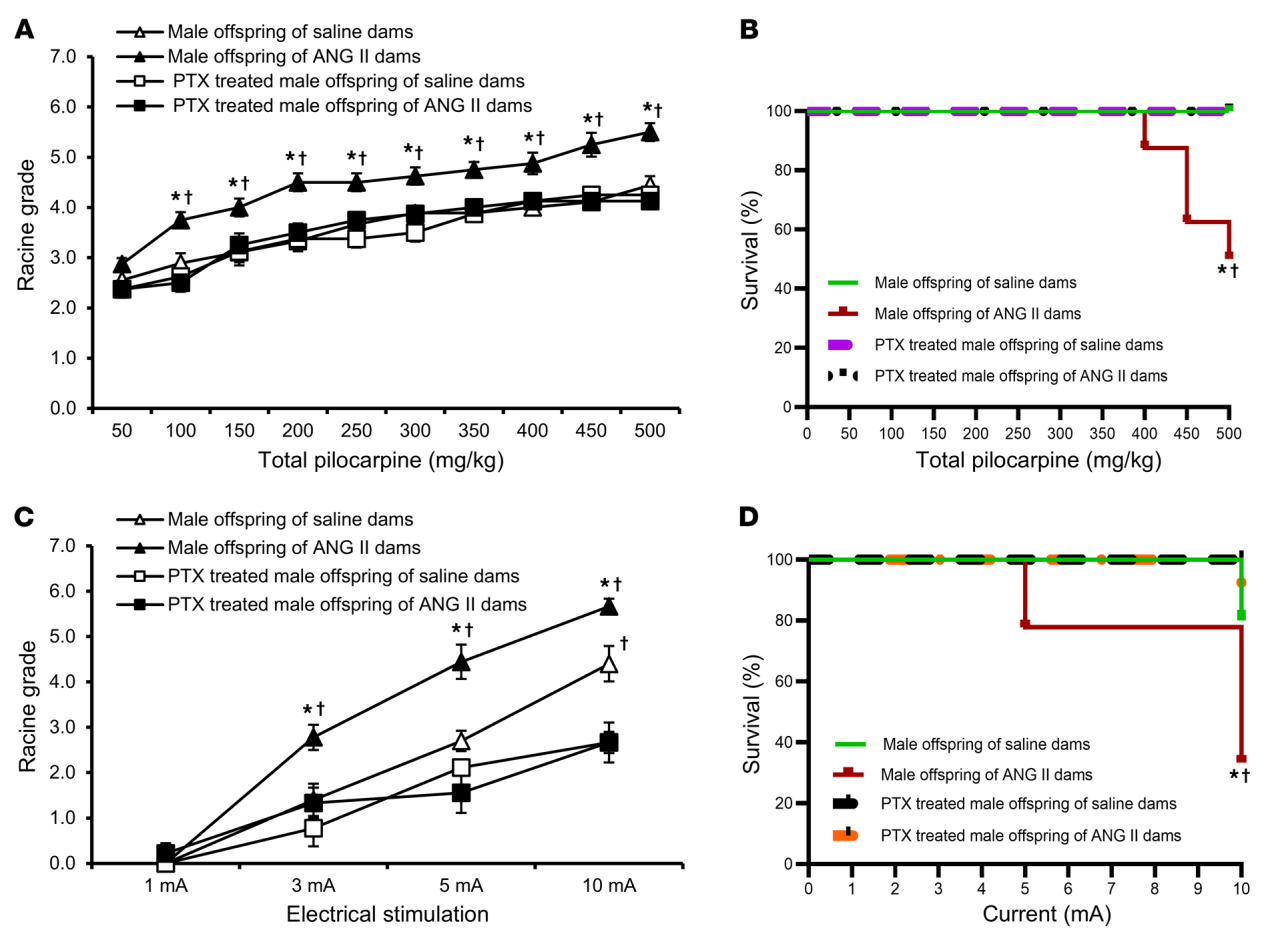
PTX rescue of male offspring seizure phenotypes. Pretreatment with PTX significantly attenuated seizure responses and prevented mortality after pilocarpine (**A** and **B**) or ES (**C** and **D**) treatment in adult male offspring of dams with ANG II–induced hypertension (*n* = 8–10/group). Two-way ANOVA followed by Tukey’s test; *P < 0.05 vs. male offspring of saline-treated dams; ^†^*P* < 0.05 vs. male offspring with PTX treatment. Data are shown as the mean ± SEM.

**Figure 6:**
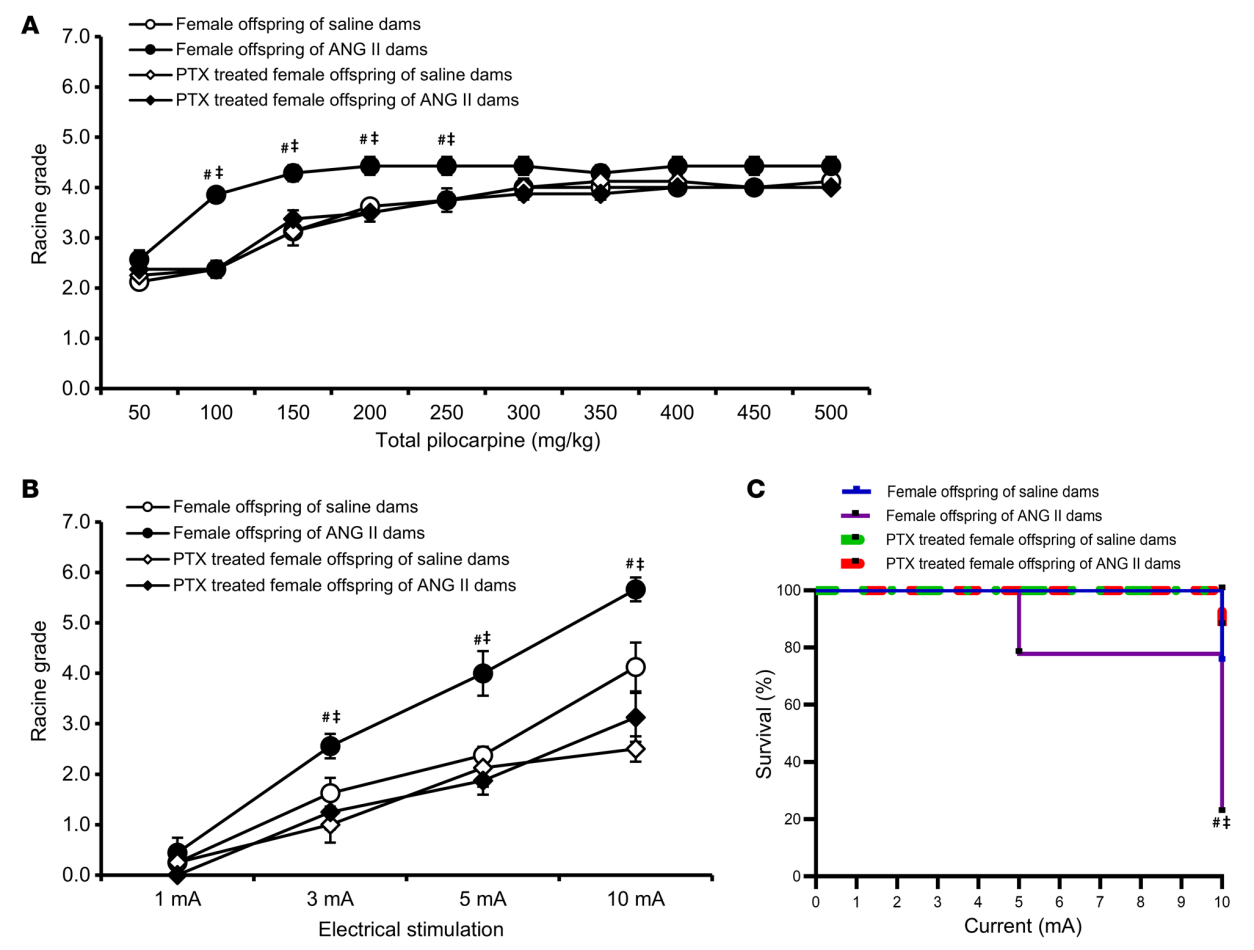
PTX rescue of female offspring seizure phenotypes. Pretreatment with PTX significantly reduced seizure responses and mortality after pilocarpine (**A**) or ES (**B** and **C**) treatment in adult female offspring of both normotensive (saline-treated) dams and dams with ANG II–induced hypertension (*n* = 7–9/group). Two-way ANOVA followed by Tukey’s test; ^#^*P* < 0.05 vs. female offspring of saline-treated dams; ^‡^*P* < 0.05 vs. PTX-treated female offspring. Data are shown as the mean ± SEM.

To determine the role of inflammation in GH-associated programming of offspring seizure susceptibility, we administered PTX to decrease proinflammatory cytokines in offspring from saline- or ANG II–treated dams. Two weeks of PTX administration via the drinking water did not change body weights for any group (Supplemental Figure 5, A and B). Pretreatment with PTX prior to seizure threshold assessment by the pilocarpine method had no effect in adult male offspring of saline-treated dams, but significantly attenuated seizure responses and prevented death following application of pilocarpine in adult male offspring of ANG II–treated dams (Figure 5, A and B). Similarly, PTX administered to offspring significantly reduced seizure responses to ES, as shown by Racine grades and mortality rates among male offspring of both saline- and ANG II–treated dams (*P* < 0.05, Figure 5, C and D). Likewise, PTX treatment also decreased seizures and mortality following pilocarpine or ES in female offspring of ANG II–treated dams (*P* < 0.05, Figure 6, A–C).

### Pharmacologic microglia depletion attenuates seizures in adult offspring of GH pregnancies.

Given the positive effects of PTX on seizure susceptibility and death in hypertension-exposed offspring, we next sought to determine the role of microglia in mediating these effects. The CSF1R inhibitor PLX5622 was administered via chow (1,200 ppm) for 1 week (32). Chow consumption and body weight were unchanged by PLX5622 administration across all groups (Supplemental Figure 5, C and D). Oral administration of PLX5622 for 7 days led to a significant depletion of microglia in the hippocampi of offspring from either saline- or ANG II–treated dams (Supplemental Figure 6, A and B). This depletion of microglia was greater in the offspring of saline-treated dams than in those of ANG II–treated dams (88.4% ± 2.3% vs 69.4% ± 3.9%, *P* < 0.05, Supplemental Figure 6C).

**Figure 7:**
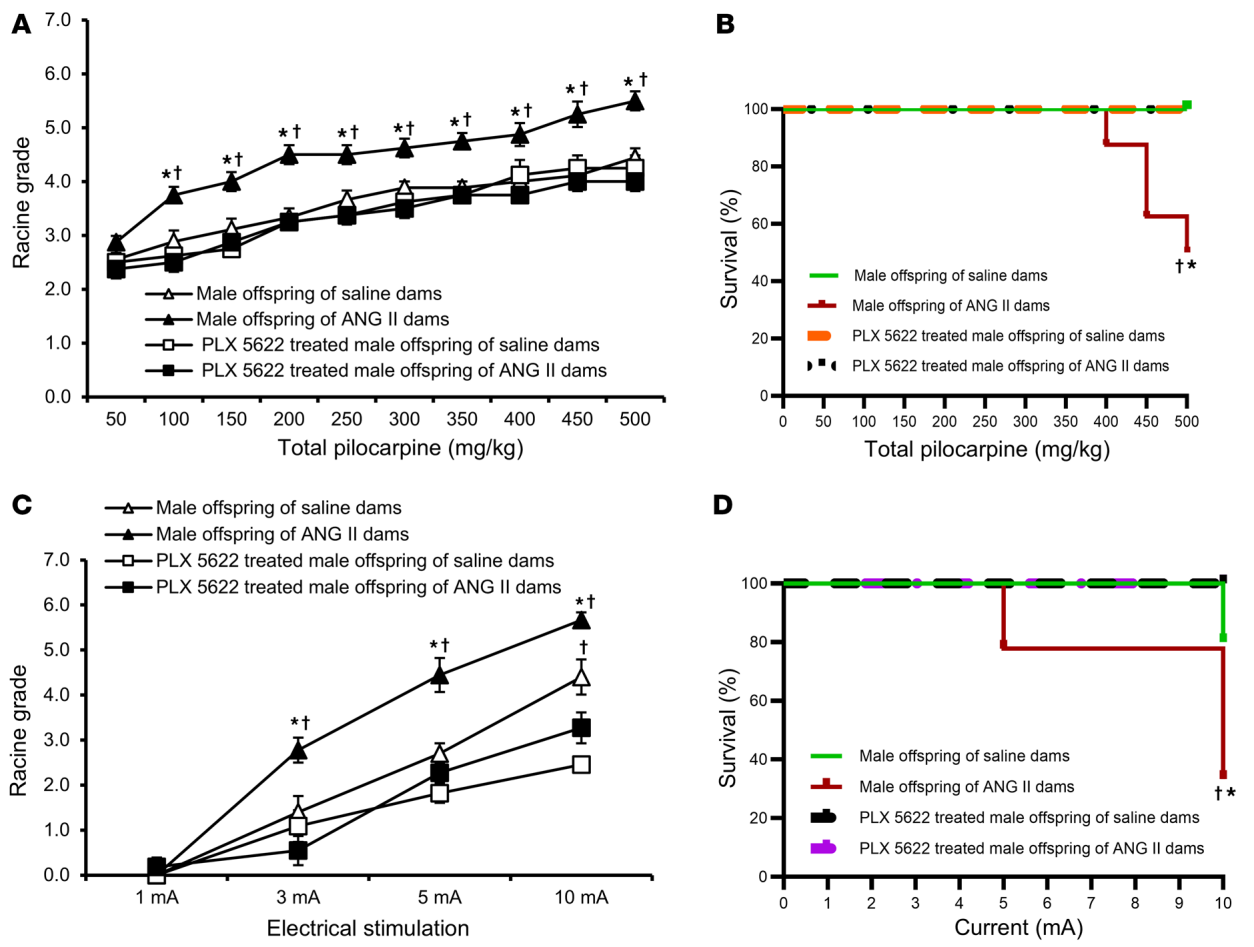
PLX5622 rescue of male offspring seizure phenotypes. Pretreatment with PLX5622 significantly attenuated seizure responses and abolished mortality after pilocarpine (**A** and **B**) or ES (**C** and **D**) treatment in adult male offspring of dams with ANG II–induced hypertension (*n* = 8–11/group). Two-way ANOVA followed by Tukey’s test; *P < 0.05 vs. male offspring of saline-treated dams; ^†^*P* < 0.05 vs. male offspring with PLX 5622 treatment. Data are shown as the mean ± SEM.

**Figure 8:**
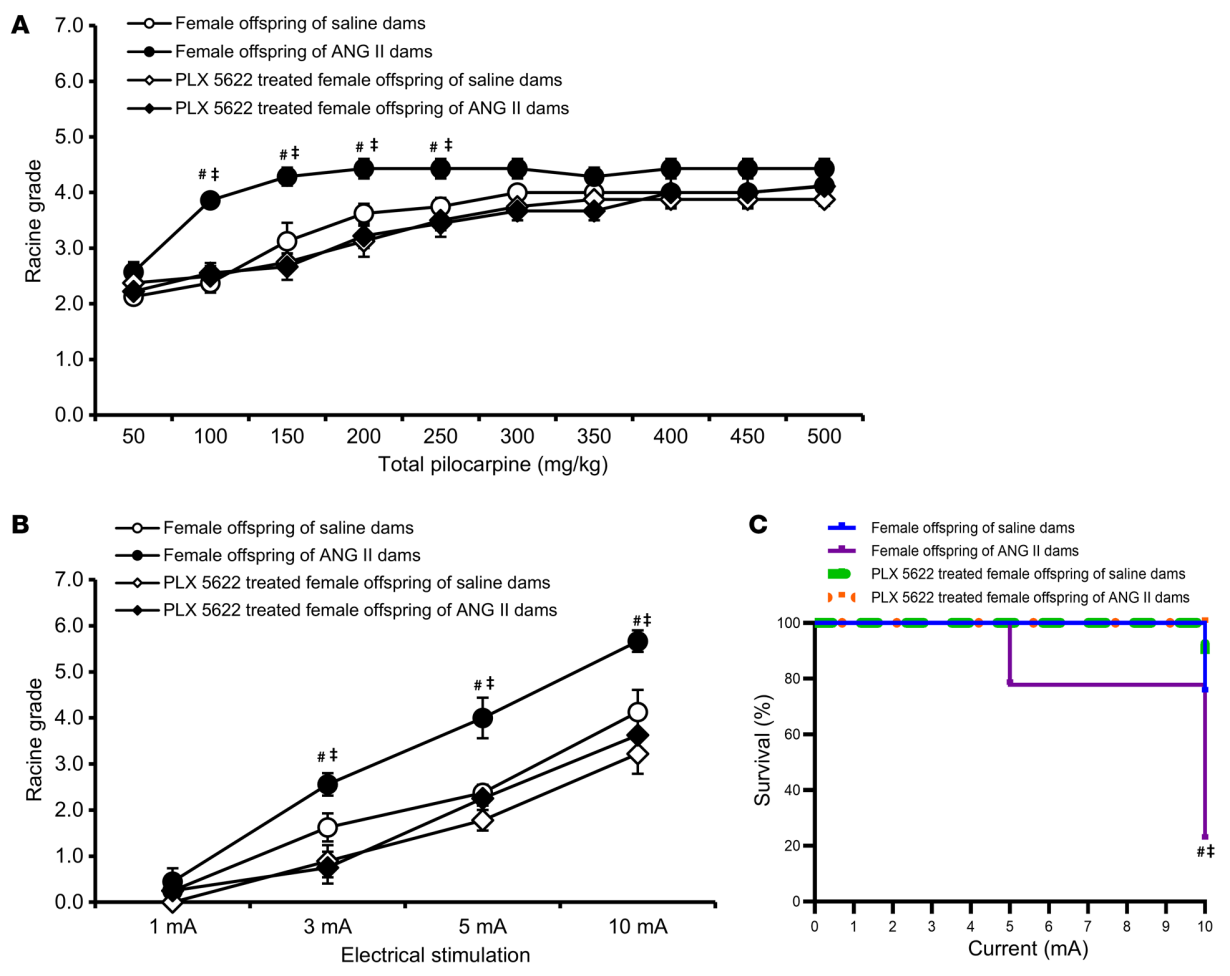
PLX5622 rescue of female offspring seizure phenotypes. Pretreatment with PLX5622 significantly reduced seizure responses and mortality after pilocarpine (**A**) or ES (**B** and **C**) treatment in adult female offspring of dams with ANG II–induced hypertension (*n* = 7–9/group). Two-way ANOVA followed by Tukey’s test; ^#^*P* < 0.05 vs. female offspring of saline-treated dams; ^‡^*P* < 0.05 vs. female offspring with PLX 5622 treatment. Data are shown as the mean ± SEM.

Although there was less depletion of microglia in the offspring of ANG II–treated dams, this microglial depletion was sufficient to significantly reduce seizure responses and abolish mortality following application of either pilocarpine or ES in both male (Figure 7) and female (Figure 8) offspring of ANG II–treated dams.

## Discussion

In this study, we utilized the large Epic Cosmos patient database (>240 million patient records) and 3 additional, complementary cohorts to assess the risk for seizure in children born to mothers who were hypertensive during pregnancy. We continued our investigation of this association using ANG II and PE infusion mouse models of GH. The present study had several major findings: (a) Children born to mothers with hypertension during pregnancy had significantly higher odds of developing a seizure than did those who were unexposed, even after controlling for covariates such as maternal BMI and age. This risk was particularly elevated among male children. (b) Both young and adult male, but not female, offspring of ANG II–treated dams exhibited upregulated proinflammatory and microglial marker expression in the hippocampus, a common seizure focus, when compared with offspring of saline-treated dams. (c) In both pharmacologic and electrical seizure induction models, young and adult offspring of ANG II–treated dams had significantly increased seizure response and death. (d) As in the ANG II model, offspring from the complementary PE GH model exhibited persistent neuroinflammatory and seizure phenotypes, but not altered *At1r* expression, suggesting that maternal hypertension was sufficient to drive prenatal seizure programming across diverse models. (e) The augmented seizure responses and death of offspring of dams with ANG II–induced hypertension were prevented by either depletion of microglia or inhibition of inflammation. Taken together, these results link exposure to hypertensive disorders during the prenatal period to increased seizure risk, seizure severity, and mortality in offspring across their lifespan. Our preclinical results further suggest that this risk was modified by neuroimmune mechanisms.

Pediatric epilepsy is a common neurological disorder with often unclear etiology (8, 33). Several small human cohort studies have suggested that preeclampsia and maternal hypertension lead to an increased risk for seizures in children (3–6) and that this risk correlates positively with the severity of preeclampsia (5–7). Our work in diverse clinical cohorts complements this epidemiology. Across the Epic Cosmos patient database, embedded University of Iowa and Stanford cohorts, and an independent, large Taiwanese insurance database, we found that children born to hypertensive mothers had a higher incidence of seizures than did those born to normotensive mothers. While Cosmos is uniquely large, it is limited in its ability to allow for line-level analyses, including covariate examination and examination of geographic trends. Complementary use of smaller cohorts allowed us to control for critical covariates.

The mechanisms underlying prenatal programming of seizure are unclear. One possibility is that dysfunction of intrauterine glucocorticoid metabolism, inflammatory mechanisms, and their interactions with the RAS may alter fetal brain development in the setting of GH (5, 6, 34). Studies have implicated RAS in preeclampsia, and ANG II probably plays an important role in the pathogenesis of preeclampsia (35, 36). Infusion of ANG II during pregnancy has been shown to mimic preeclampsia/maternal hypertension-like phenotypes in rats, including increased BP, albuminuria, shallow trophoblast invasion accompanied by a reduced percentage of remodeled spiral arteries in the decidua, systemic inflammation, and reduced placental perfusion and intrauterine growth restriction (35, 37–39). These phenotypes likely reflect the broad pathophysiologic and hemodynamic effects of ANG II, including increased BP, enhanced ER stress, oxidative stress, and proinflammation (25, 27, 40). Here, we have further implicated neuroimmune processes in GH programming of offspring seizure disorder risk by virtue of the results we observed in the ANG II model. We report that maternal ANG II was sufficient to cause neuroinflammation and increased seizure sensitivity, severity, and death in offspring. Either the presence of microglial or broader brain inflammation in offspring was necessary for this seizure phenotype.

Given the effects of ANG II on multiple putative seizure risk pathways, including nonspecific inflammatory and hemodynamic mechanisms, we complemented ANG II studies with another hypertension model — the chronic PE model. PE, an α1 adrenergic receptor agonist, does not cause the same chronic proinflammatory and pathophysiologic effects in dams as chronic ANG II (25, 27, 40). Despite the markedly different mechanisms of action by which ANG II and PE induce GH, both PE offspring and ANG II offspring exhibited persistent seizure programming.

Our preclinical results are consistent with those obtained with other preeclampsia and maternal hypertension models, for example, those induced by transgenic means (indoleamine 2,3-dioxygenase knockout) or by infusion of vasopressin or *N*(ω)-nitro-l-arginine methyl ester (34, 35, 41–44). An important element of these models, which we will examine in future work, is that they also result in metabolic reprogramming of offspring and intrauterine growth restriction or reduced birth weight, as we report in the GH models in this study. This reduction in offspring weight may itself increase offspring neurologic risk (45, 46).

Seizure risk is associated with neuronal damage, gliosis, microgliosis, and increased levels of inflammatory factors in the microenvironment of neural tissues (14). Activated glia produce inflammatory cytokines within 4 hours of seizure induction in animal models and in human chronic epilepsy tissues (17). Moreover, previous studies showed that microglia recruitment and activation can decrease seizure thresholds by triggering the production of proinflammatory cytokines with neuromodulatory properties (47). TNF is a highly conserved proinflammatory cytokine that initiates the release of other cytokines (e.g., IL-1β and IL-6), increases glutamate receptor numbers, and induces GABA receptor endocytosis, leading to increased excitatory drive (48). Liu and colleagues reported that l-NAME–induced maternal hypertension disrupts offspring brain development, increasing gliogenesis and reducing neurogenesis, leading to cognitive defects (34). In the present study, we also found upregulation of the neuroinflammatory markers *Tnf* and *Cd11b* in the hippocampi of male, but not female, young and adult offspring from hypertensive dams prior to any seizure challenge. Neuroinflammation may represent a sensitizing mechanism, predisposing offspring to the development of seizures in response to secondary hits later in life. We and others reported previously that offspring of the arginine vasopressin or ANG II animal models for the study of preeclampsia and maternal hypertension exhibit dysregulation of immune-related genes in the brain (2, 49–51). However, here, we extended these results to demonstrate that GH increased microglia activation and neuroinflammation and sensitized the seizure response.

Our findings highlight a role for the neuroimmune system in GH-mediated programming of seizure disorder risk in offspring. This was further confirmed by administration of drugs to offspring to deplete microglia and inhibit proinflammation before seizure stimulation. Either depletion of microglia or inhibition of inflammation in both male and female offspring of hypertensive dams abolished their sensitized seizure response and death. The results indicate that disruption of offspring microglial and inflammatory responses to GH may affect neural activity in the seizure-generating hippocampus.

Our preclinical work across 2 models of GH revealed an intriguing sex-specific vulnerability of male offspring to neuroinflammation and seizure after exposure to GH. Many components of neuroinflammation are linked to sexually dimorphic mechanisms, including hormonal and immunological processes (52). Sex differences upstream of inflammation may also contribute to sex differences in seizure. For instance, there are noted sex differences in hippocampal neuroimmune reactivity, a state that may create a brain milieu that is proinflammatory and favorable to seizure (53).

Unlike pilocarpine-induced sex differences in seizures, ES induced similar enhancements in the seizure response and death in both sexes and in both young and adult offspring from hypertensive dams. Diazepam rescue confirmed that death after ES was in fact caused by seizures. The divergent results we observed with pilocarpine and ES may be the consequence of their differing seizure propagation patterns. ES via transauricular electroshock (ear-clip electrodes) induces seizure through activation of the brainstem, whereas pilocarpine elicits seizure by binding to muscarinic type 1 receptors, particularly in the hippocampus, to enhance neuronal excitability (54). As components of tonic seizures depend on the brainstem, transauricular stimulation elicits tonic convulsions, which are more severe and present lower and less variable seizure thresholds, more effectively than other approaches (55–57). Given the significantly reduced seizure threshold in offspring of hypertensive dams, we speculate that transauricular stimulation used in the present study may have eliminated the age- and sex-related difference in the sensitivity to seizures induced by maternal hypertension.

Our study illuminates exciting future directions for research on the link between GH and seizure risk in offspring. In the current study, we focused on GH-induced alterations in the hippocampus because of the robust literature linking hippocampal dysregulation to pediatric seizure disorders. However, the hippocampus has direct connections to other brain regions that are also important for the development of epilepsy, such as the amygdala and the bed nucleus of the stria terminalis (BNST) (58). How maternal hypertension modifies the neurodevelopment of these other structures and their connections is an important question for the future. Future studies should also examine the potential roles of a variety of brain cell types. In addition to microglia, neurons, astrocytes, and diverse neuroendocrine systems (e.g., the RAS, serotonin) are involved in seizure and epileptogenesis (14, 31, 59, 60). The intriguing sex differences we report here also merit further investigation. Ovarian and testicular function can be altered by an adverse uterine environment (61), and progesterone, estrogen, and androgens all can affect seizure susceptibility (62). Our results raise the possibility that sex hormones modulate long-term changes in seizure risk in response to a prenatal insult, and this needs to be evaluated. Future work should also interrogate and compare the prenatal and postnatal effects of GH, as maternal hypertension oftentimes does not resolve with delivery but carries into the postnatal period with effects on lactation and parental care (63, 64).

GH is a complex exposure, involving multiple maternal mechanisms (e.g., oxidative stress, inflammation, antiangiogenesis, etc.) and interacting sequelae for children such as low birth weight, increased infection risk, prematurity, and more. Future work in large clinical datasets should seek to disentangle these features to determine the primary and interacting drivers of seizure risk. The rates of pediatric seizure reported here, as in other clinical datasets, are likely higher than might be expected in the general population due to selection and morbidity biases (65). However, the increased seizure disorder risk with GH that we report was seen across multiple large cohorts and after adjusting for several important covariates. Epidemiologic data that capture nonclinical populations may help reduce the risk of morbidity and selection biases seen in electronic health record–derived samples, such as those used here and would provide a means to refine our quantitation of GH-associated seizure disorder risk. Finally, determining the placenta/brain axis mechanisms of fetal brain programming remains a looming question in the field and must be better understood to advance early biomarkers, prophylactics, and cures (66).

In summary, by utilizing the enormous sample size of the Epic Cosmos dataset, granular data from the Iowa- and Stanford-based cohorts, and a large international cohort from Taiwan, the present study demonstrates an association between hypertension of all causes during pregnancy and seizures in children. We also found that 2 distinct, but complementary, mouse models of GH were sufficient to cause increased rates of seizure and seizure-associated death in the offspring. Each of these complementary models invoked a different underlying pathology but both were sufficient to cause GH and altered neuroimmunity in the offspring. Sex-dependent differences in GH seizure programming were found in humans and mice. The protective effects of microglial depletion and inhibition of inflammation on offspring seizure phenotypes suggest that GH-induced seizure sensitization may be reversed by interruption of inflammatory pathways, opening a novel and powerful therapeutic window preceding seizure onset.

## Methods

### Sex as a biological variable.

Our study examined both males and females in experimental models and human datasets, including differences between male and female mice and humans.

Additional details on methods can be found in the Supplemental Methods.

### Epic Cosmos study.

The human data used in this study were obtained from the Epic Cosmos dataset, representing over 246 million patient records from over 1,400 hospitals and 33,000 clinics in the United States and Lebanon, including 8 million patients with birth parent information. This dataset encompasses a wide array of information beyond standard diagnoses and medications, such as patient-generated health data, birth records, vital signs, and social determinants of health.

The time frame for Epic Cosmos data analysis spanned from January 1, 2011, to December 31, 2023. We included patients who met the definition of “base patient” (more than one face-to-face encounter with a health care provider in any 2-year interval). We included the following diagnosis sources: encounter diagnosis, billed final diagnosis, billed charge-associated diagnosis, billed admitting diagnosis, and admitting diagnosis. This selection criterion was implemented to enhance the precision of our analysis by excluding cases where hypertension was only recorded on the problem list. Maternal and child conditions were identified using the International Classification of Diseases, Tenth Revision (ICD-10) codes and Systemized Nomenclature of Medicine Clinical Terms (SNOMED CT). Maternal hypertension was captured using the codes ICD-10-CM: O10 (preexisting hypertension complicating pregnancy, childbirth, and the puerperium); ICD-10-CM: O11 (preexisting hypertension with preeclampsia); ICD-10-CM: O13 (gestational[pregnancy-induced] hypertension without significant proteinuria); ICD-10-CM: O14 (preeclampsia); ICD-10-CM: O16 (unspecified maternal hypertension); ICD-10-CM: I10 (essential [primary] hypertension); and ICD-10-CM: I15 (secondary hypertension), while child epilepsy and seizures were identified with the codes ICD-10-CM: G40 (epilepsy and recurrent seizures); ICD-10-CM: R56 (convulsions, not elsewhere classified); ICD-10-CM: P90 (convulsions in newborn); ICD-10-CM: R25.9 (unspecified abnormal involuntary movements); and ICD-10-CM: R40.4 (transient alteration of awareness). The SNOMED CT codes were used to identify 3 maternal and/or child conditions concomitant with maternal hypertension and child seizures, respectively: diabetes mellitus (code 73211009), obesity (code 414915002), and developmental delay (code 248290002).

### IGHK study.

This case-control study uses a dataset from the Intergenerational Health Knowledgebase (*n* = 78,726 pregnancies, IRB no. 20101369), an integrated data platform for all short-term and long-term electronic health records (EHR) data on maternal, pediatric, and pregnancy care at the University of Iowa Health Care (UIHC) system. Composite case definitions of neonatal seizures (G40, R56, P90, R25.9, R40.4) and HDP (O10–11, O13–14, O16, I10, I15) was constructed using ICD-10 codes. Baseline characteristics were compared between cases and controls (α = 0.05). Logistic regression models were constructed to evaluate the association between the development of neonatal seizures and HDP.

### Stanford validation cohort study.

Mothers and their children were identified from STARR–Observational Medical Outcomes Partnership (STARR-OMOP), which is the Stanford Electronic Health Records research database. STARR-OMOP data are represented in the OMOP Common Data Model (CDM), which is a widely used data model for representing observational health data (67). STARR-OMOP contains data from both the adult Stanford Healthcare system and the pediatric Lucile Packard Children’s Hospital system. EHR data on mothers and their linked children were identified from September 9, 1993, to September 22, 2024. Mother and child linkages were identified from the OMOP Fact Relationship Table using the relationship concept IDs of 581426 and 581437. Only patients with at least 2 face-to-face encounters in any 2-year period were included. Demographics (age, sex, ancestry) and diagnosis codes were identified from the EHR. The ICD and SNOMED CT codes used to identify diagnoses in the Cosmos and IGHK databases were converted to OMOP standard codes. All codes used to identify diagnoses also included their descendants in the ontological hierarchy.

The primary predictor was maternal hypertension, identified by at least 1 occurrence of the following conditions during pregnancy: preexisting hypertension complicating pregnancy, childbirth, and the puerperium (ICD10-CM O10, OMOP 321074); preexisting hypertension with preeclampsia (ICD10-CM O11, OMOP 4283352); gestational (pregnancy-induced) hypertension without significant proteinuria (ICD-10-CM O13, OMOP 4167493); preeclampsia (ICD-10-CM O14, OMOP 439393); unspecified maternal hypertension (ICD-10-CM O16, OMOP 4118910); essential (primary) hypertension (ICD-10-CM I10, OMOP 320128); and secondary hypertension (ICD-10-CM I15, OMOP 319826).

The primary outcome was child epilepsy and seizures, identified with at least 1 occurrence before the age of 18 of the following events or conditions: epilepsy and recurrent seizures (ICD-10-CM G40, OMOP 380378 and 377091); convulsions, not elsewhere classified (ICD-10-CM R56, OMOP 377091); convulsions in the newborn (ICD-10-CM P90, OMOP 380533); unspecified abnormal involuntary movements (ICD-10-CM R25.9, OMOP 45539328 and 376229); transient alteration of awareness (ICD-10-CM R40.4, OMOP 37160989 and 4275359).

To identify maternal and child conditions concomitant with maternal hypertension and child seizures, respectively, the following codes were used: maternal diabetes mellitus (SNOMED CT 73211009, OMOP 436077); maternal obesity (SNOMED CT 414915002, OMOP 433736); and child developmental delay (SNOMED CT 248290002, OMOP 436077).

Basic descriptive analyses were performed as well as calculation of ORs for child seizure for those with and without maternal hypertension. These statistical analyses were performed in Python (version 3.10.14) using the package statsmodels (version 0.14.2). A multivariable logistic regression was also performed to evaluate the odds of child seizures of offspring from mothers with hypertension compared with mothers without hypertension, adjusted for maternal age, BMI, diabetes, and child ancestry. This analysis was performed in R (version 4.4.2).

### International validation cohort study.

Our international validation, population-based study utilized the National Health Insurance Research Database (NHIRD) of Taiwan, which contains insurance claims data on beneficiaries under the National Health Insurance (NHI) Program. These data cover up to 99.9% of Taiwan’s population (68). To enhance and centralize available data, Taiwan’s Ministry of Health and Welfare established the Health and Welfare Data Center (HWDC) to link the NHIRD with other health-related databases, including the Maternal and Child Health Database and the Birth Registry Database. Here, we linked maternal-child records from the Maternal and Child Health Database and the NHIRD Birth Registry Database for the years 2000 to 2015. Eligible children were required to have at least 2 follow-up visits within any 2-year interval before reaching 18 years of age to ensure adequate health monitoring. Stillbirths and multiple births from the same delivery were excluded.

The primary exposure of interest was maternal hypertension during pregnancy, which included both preexisting hypertension and GH. Pregnancy duration was determined on basis of the gestational weeks recorded in the Birth Registry Database. The primary outcome was a childhood seizure diagnosis before 18 years of age. Maternal hypertension and childhood seizures were identified using the relevant ICD-9 and ICD-10 diagnosis codes listed in Supplemental Table 11. Covariates included maternal age at delivery from the Birth Registry Database and diabetes mellitus and obesity status, as defined by the appropriate ICD-9 and ICD-10 diagnosis codes (Supplemental Table 11). Delivery-related covariates included gravida, gestational weeks, infant’s sex, birth weight, delivery method (e.g., vaginal delivery or Cesarean section), Apgar scores, congenital defects, delivery complications, and pregnancy procedures as recorded in the Birth Registry Database.

For this cohort’s descriptive analyses, categorical variables are presented as frequency (proportion) and analyzed using the χ^2^ test, whereas the continuous variables are presented as the median (first and third quantile) and analyzed using the Wilcoxon rank-sum test. The association of maternal hypertension with the risk of childhood seizure was investigated using both a multivariate logistic regression model and a Cox proportional hazards model, adjusting for maternal variables of age, diabetes, obesity, and gestational weeks, and infant variables of birth weight, fifth-minute Apgar score, and developmental delay occurring before the seizure event. In the Cox model, a competing risk analysis was performed using a cause-specific Cox proportional hazards model, with death regarded as the censoring event. A 2-sided α value of 0.05 indicated statistical significance. All statistical analyses were performed using SAS (version 9.4, SAS Institute) in a secured area of the Data Science Center, Ministry of Health and Welfare, Taiwan.

### Animal studies.

Eighty female and 40 male mice (C57Black/6J, 10 weeks old, The Jackson Laboratory) were used for breeding. The dams were instrumented with telemetry probes (TA11PA-C10, Data Sciences International) through the carotid artery for continuous monitoring of BP and HR. After baseline BP and HR recordings were made, female dams were implanted with subcutaneous osmotic minipumps (Alzet, no. 1004) loaded with vehicle (saline), ANG II (s.c., 1,200 ng/kg/min, 4 weeks), or PE (s.c., 60 μg/kg/min, 4 weeks), which delivered their contents continuously throughout mating and pregnancy. Offspring were weighed and counted at birth, and both male and female offspring were used in all experiments. Experimental animals were randomly selected and balanced across litters.

At 10–12 weeks (adult) of age, a subset of male and female offspring from normotensive (saline) or hypertensive (ANG II or PE) dams were euthanized, and brains were collected for molecular and histologic analyses (*n* = 5–6/group). Another subset of offspring from saline- or ANG II–treated dams that had received PTX in the drinking water or PLX 5622 in the chow were also euthanized and processed in the same way to assess hippocampal inflammation (*n* = 5–6/group).

In separate experiments, at 10–12 weeks of age, male and female offspring of saline-, ANG II–, and PE-treated dams were used to determine whether maternal hypertension during pregnancy resulted in an increased risk of seizure and to evaluate the effects of antiinflammatory treatments or microglial depletion on seizure risk. Chemical seizure induction experiments included a total of 7 groups (*n* = 7–9/group/sex): (a) offspring of saline-treated dams with pilocarpine, (b) offspring of ANG II–treated dams with pilocarpine, (c) PTX-treated offspring of saline-treated dams with pilocarpine, (d) PTX offspring of ANG II–treated dams with pilocarpine, (e) PLX5622 offspring of saline-treated dams with pilocarpine, (f) PLX5622 offspring of ANG II–treated dams with pilocarpine, (g) offspring of PE-treated dams with pilocarpine. The sexes were tested separately in all cases. For experiments using ES, adult male and female offspring were divided into 9 groups (*n* = 7–11/group/sex): (a) offspring of saline-treated dams with ES, (b) offspring of ANG II–treated dams with ES, (c) diazepam-treated offspring of saline-treated dams with ES, (d) diazepam-treated offspring of ANG II–treated dams with ES, (e) PTX offspring of saline-treated dams with ES, (f) PTX offspring of ANG II–treated dams with ES, (g) PLX5622 offspring of saline-treated dams with ES, (h) PLX5622 offspring of ANG II–treated dams with ES, (i) offspring of PE-treated dams with ES. The sexes were tested separately in all cases.

### Diazepam, PTX or PLX 5622 treatment.

Diazepam (5 mg/kg, i.p.) was given to offspring 30 minutes before ES. At 8 weeks of age, offspring from saline or ANG II dams were given PTX (1.3 mg/mL, MilliporeSigma) in their drinking water for 2 weeks, which resulted in a PTX dose of 150 mg/kg/day. At 9 weeks of age, the offspring were provided ad libitum access to PLX5622 or vehicle diet for 7 days. PLX5622 was formulated in AIN-76A rodent chow (Research Diets) at a concentration of 1,200 ppm. A standard AIN-76A diet was provided as a vehicle control.

### Induction of chemical seizures and electrical seizures.

Chemical seizures were induced by consecutive injections of pilocarpine (50 mg/ kg, i.p.) at an interval of 20 minutes until a cumulative dose of 500 mg/kg was achieved. Electrical seizures were elicited by increasing the current (1, 3, 5, and 10 mA; 5 minutes between stimulations) until seizures and/or death occurred.

A modified Racine scale was used to characterize behavioral seizures, as described previously (31): 0, behavioral arrest; 1 and 2 (grouped together), facial automatisms, tremor, tail stiffening, head bobbing, body jerks; 3, single limb myoclonus; 4, bilateral myoclonus, rearing, nonsustained tonic-clonic activity; 5, recurrent (<2 minutes apart), sustained tonic-clonic seizures (status epilepticus) and/or extension; 6, death.

### Quantitative real-time PCR.

Quantitative comparison of mRNA expression of inflammatory and microglial markers [*Tnf* (Mm00443258), *Cd11b* (Mm00434455)] was determined by TaqMan PCR in the hippocampus in offspring from saline, ANG II, and PE dams. Genes of interest were normalized to the endogenous control *Gapdh* (Mm99999915). The final concentration of mRNA was calculated using the formula *x* = 2^(−ΔΔCt)^, and results are expressed as the fold difference from saline controls.

### Statistics.

For analyses of human data from the Epic Cosmos dataset, the χ^2^ contingency test with Yates correction was applied. ORs and corresponding 95% CIs were calculated. In animal studies, MAP, HR, and locomotor activity are presented as mean daily values. Two-way ANOVAs by condition were conducted on daily MAP, HR, or activity, and significant ANOVAs were followed by a post hoc Tukey’s multiple-comparison test. The same statistical approach was used for Racine scale results. Offspring survival after application of pilocarpine or ES was analyzed by log-rank (Mantel-Cox) test. One-way ANOVAs with a post hoc Tukey’s multiple-comparison or Kruskal-Wallis test, followed by Dunn’s multiple-comparison test (non-normally distribution data) were used to compare differences in litter size, pup birth weight, and gene expression between groups. All data are expressed as the mean ± SEM. Statistical significance was set at a *P* value of less than 0.05. Outliers were tested using the “identify outliers” function in GraphPad Prism 10.4.1 (GraphPad Software) and were removed.

### Study approval.

All experimental protocols were approved by the IACUC of the University of Iowa. The University of Iowa IRB reviewed the research protocol and determined that the use of the Epic Cosmos database did not constitute human subject research, as it falls under Federal Exemption 4. This study was approved by the IRB of China Medical University Hospital (CMUH111-REC2-155).

### Data availability.

Values for all data points in graphs are reported in the Supporting Data Values file.

## Author contributions

AGB, BX, JMM, and SBG conceived and designed the study. BX, GT, BPT, AW, JMM, CCK, HYC, RR, SYW, and HAD performed experiments. BX, AGB, and SBG drafted and revised the manuscript. DAS, MKS, VBM, AKJ, PJF, JMM, SBG, and EAN reviewed and edited the manuscript.

## Supplementary Material

Supplemental data

Supporting data values

## Figures and Tables

**Table 4:**
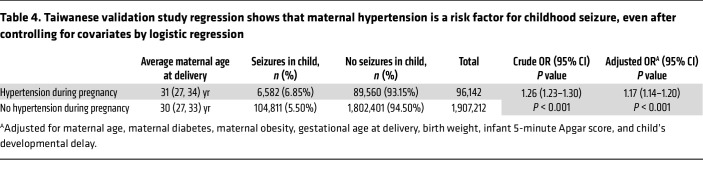
Taiwanese validation study regression shows that maternal hypertension is a risk factor for childhood seizure, even after controlling for covariates by logistic regression

**Table 3:**
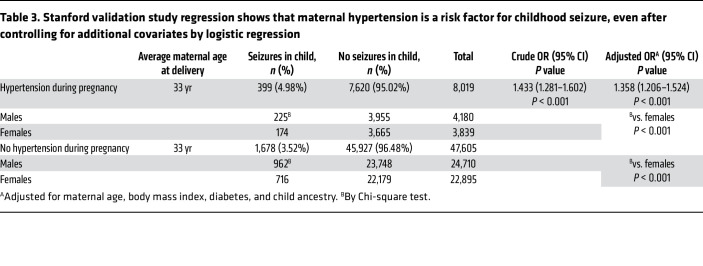
Stanford validation study regression shows that maternal hypertension is a risk factor for childhood seizure, even after controlling for additional covariates by logistic regression

**Table 2:**
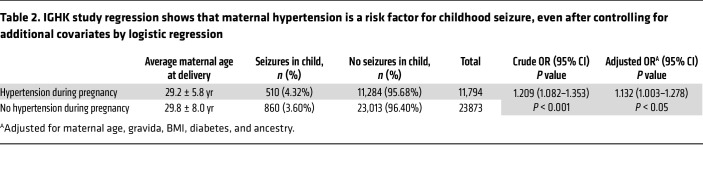
IGHK study regression shows that maternal hypertension is a risk factor for childhood seizure, even after controlling for additional covariates by logistic regression

**Table 1:**
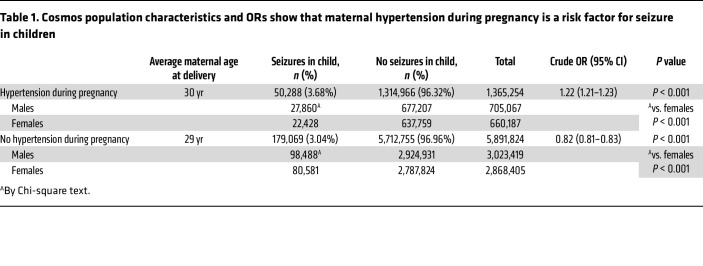
Cosmos population characteristics and ORs show that maternal hypertension during pregnancy is a risk factor for seizure in children

## Version 1 06/16/2025

Electronic publication
