# Supplementary material for: Gestational hypertension increases risk of seizures in children and mice
Source: J Clin Invest. 2025 Jun 16;135(12):e183393. doi: 10.1172/JCI183393 (PMC12165801; doi:10.1172/JCI183393)

Online Supplement

Gestational Hypertension Increases risk of Epilepsy in Children and in Mice

Baojian Xue[1], Serena B Gumusoglu[2,3,4], Grant Tiarks[1], Brittany P Todd[1], Angela Wong[1], Donna A Santillan[2,3,4], Chin-Chi Kuo[5,6,7], Hsiu-Yin Chiang[5,6], Rohith Ravindranath[8], Sophia Y. Wang[8], Vinit Mahajan[9], Alan Kim Johnson[10], Heath Davis[11], Polly J Ferguson[1], Elizabeth A Newell[1], Mark K Santillan[2,3,4], Jason M Misurac[1], Alexander G Bassuk[1,12,13]

Stead Family Department of Pediatrics[1], Department of Obstetrics and Gynecology[2], Department of Psychiatry[3], the Hawk IDDRC[4], Roy J. and Lucille A. Carver College of Medicine, University of Iowa, Iowa City, IA 52242, USA
Big Data Center[5], Department of Biomedical Informatics[6], Department of Internal Medicine[7], China Medical University Hospital, Taichung, Taiwan
Byers Eye Institute[8], Molecular Surgery Laboratory[9], Stanford University, Palo Alto, CA 94304, USA
Department of Psychological and Brain Sciences[10], University of Iowa, Iowa City, IA, USA
Institute for Clinical and Translational Science-Biomedical Informatics[11], University of Iowa, Iowa City, IA, USA

The Iowa Neuroscience Institute[12] and The Department of Neurology[13], Roy J. and Lucille A. Carver College of Medicine, University of Iowa, Iowa City, IA 52242, USA

Running head

Gestational Hypertension and Offspring Seizure

Corresponding Authors

Alexander G. Bassuk, MD and PhD

Stead Family Department of Pediatrics

The University of Iowa

25 S. Grand Ave, 2040 Medical Laboratories,

Iowa City, IA 52242-1009

Email: alexander-bassuk@uiowa.edu

Results

Maternal ANG II-induced hypertension increased long-term seizure frequency in offspring

All seizures were verified against the power spectrum (high-frequency gamma bands), by electromyogram, and against videos synchronized to EEG, as well as via a MATLAB-based algorithm. At week 10 after pilocarpine injection, offspring of angiotensin (ANG) II dams exhibited an increased seizure frequency (3.3±0.8, $p<0.05$, **Supplemental Figure 7**) when compared to offspring of saline dams (1.3±0.6)

Methods

All experiments were conducted in accordance with the NIH *Guide for the Care and Use of Laboratory Animals* and approved by the University of Iowa Animal Care and Use Committee.

Pharmacological agents

Drugs were obtained from the following sources: Angiotensin II, Sigma (Cat. A9525); Phenylephrine hydrochloride, Sigma (Cat. P6126); Pentoxifylline, Sigma (Cat. P1784); Diazepam, Sigma, (Cat. D-907); PLX5622, Chemgood (Cat. C-1521); scopolamine methyl nitrate, Sigma (Cat. S8502); pilocarpine, Sigma (Cat. P6503).

Human studies

Epic Cosmos study

Human data used in this study came from the Epic Cosmos dataset, representing over 246 million patient records from over 1,400 hospitals and 33,000 clinics in the United States and Lebanon, including 8 million patients with birth parent information. This dataset encompasses a wide array of information beyond standard diagnoses and medications, such as patient-generated

health data, birth records, vitals, and social determinants of health. The University of Iowa Institutional Review Board (IRB) reviewed the research protocol and determined that the use of the Epic Cosmos database did not constitute human subject research as it falls under Federal Exemption 4.

The timeframe for Epic Cosmos data analysis spanned from January 1, 2011, to December 31, 2023. We included patients who met the definition of "base patient" (more than one face-to-face encounter with a health care provider in any two-year interval). We included the following diagnosis sources: encounter diagnosis, billed final diagnosis, billed charge-associated diagnosis, billed admitting diagnosis, and admitting diagnosis. This selection criterion was implemented to enhance the precision of our analysis by excluding cases where hypertension was only recorded on the problem list. Maternal and child conditions were identified using the International Classification of Diseases, Tenth Revision (ICD-10) codes and Systemized Nomenclature of Medicine Clinical Terms (SNOMED CT). Maternal hypertension was captured using codes ICD-10-CM: O10 (Pre-existing hypertension complicating pregnancy, childbirth, and the puerperium), ICD-10-CM: O11 (Pre-existing hypertension with pre-eclampsia), ICD-10-CM: O13 [Gestational (pregnancy-induced) hypertension without significant proteinuria], ICD-10-CM: O14 (Pre-eclampsia), ICD-10-CM: O16 (Unspecified maternal hypertension), ICD-10-CM: I10 [Essential (primary) hypertension], and ICD-10-CM: I15 (Secondary hypertension), while child epilepsy and seizures were identified with codes ICD-10-CM: G40 (Epilepsy and recurrent seizures), ICD-10-CM: R56 (Convulsions, not elsewhere classified), ICD-10-CM: P90 (Convulsions of newborn), ICD-10-CM: R25.9 (Unspecified abnormal involuntary movements), and ICD-10-CM: R40.4 (Transient alteration of awareness). The SNOMED CT codes were used to identify maternal and child conditions concomitant with maternal hypertension and child

seizures, respectively: Diabetes mellitus (73211009), Obese (414915002) and Developmental delay (248290002).

Iowa Intergenerational Health Knowledgebase study

This case-control study uses a dataset from the Intergenerational Health Knowledgebase (n=78,726 pregnancies, IRB#20101369) an integrated data platform of all short-term and long-term electronic health records (EHR) data of maternal, pediatric, and pregnancy care at the University of Iowa Healthcare (UIHC) system. Composite case definitions of neonatal seizures (G40, R56, P90, R25.9, R40.4) and HDP (O10-11, 13-14, 16, I 10,15) were constructed using ICD-10 codes. Baseline characteristics were compared between cases and controls (α=0.05). Logistic regression models were constructed to evaluate the association between the development of neonatal seizures and HDP.

Stanford validation cohort study

Mothers and their children were identified from STARR-OMOP, which is the Stanford Electronic Health Records research database (*https://med.stanford.edu/starr-omop.html*). STARR-OMOP data is represented in the Observational Medical Outcomes Partnership (OMOP) Common Data Model (CDM) (*https://ohdsi.github.io/CommonDataModel/dataModelConventions.html; https://ohdsi.github.io/CommonDataModel/dataModelConventions.html*), which is a widely used data model for representing observational health data (1). STARR-OMOP contains data from both the adult Stanford Healthcare system (SHC) and the pediatric Lucile Packard Children's Hospital (LPCH) system. EHR data of mothers and their linked children were identified from September 9th, 1993, to September 22, 2024. Mother and child linkages were identified from the

OMOP *fact relationship* table using the relationship concept IDs of 581426 and 581437. Only patients with at least two face-to-face encounters in any 2-year period were included. Demographics (age, gender, ancestry, ethnicity) and diagnosis codes were identified from the EHR. The International Classification of Disease and SNOMED CT codes used to identify diagnoses in the Cosmos and IGHK database were converted to OMOP standard codes (*https://ohdsi.github.io/CommonDataModel/vocabulary.html*). All codes used to identify diagnoses also included their descendants in the ontological hierarchy.

The primary predictor was maternal hypertension, identified by at least one occurrence of the following conditions during pregnancy: Pre-existing hypertension complicating pregnancy, childbirth, and the puerperium (ICD10-CM O10, OMOP 321074); Pre-existing hypertension with pre-eclampsia (ICD10-CM O11, OMOP 4283352); Gestational (pregnancy-induced) hypertension without significant proteinuria (ICD-10-CM O13, OMOP 4167493); Pre-eclampsia (ICD-10-CM O14, OMOP 439393); Unspecified maternal hypertension (ICD-10-CM O16, OMOP 4118910); Essential (primary) hypertension (ICD-10-CM I10, OMOP 320128); and Secondary hypertension (ICD-10-CM I15, OMOP 319826).

The primary outcome was child epilepsy or seizures, identified with at least one occurrence before the age of 18 of the following conditions: Epilepsy and recurrent seizures (ICD-10-CM G40, OMOP 380378, 377091); Convulsions, not elsewhere classified (ICD-10-CM R56, OMOP 377091); Convulsions of newborn (ICD-10-CM P90, OMOP 380533); Unspecified abnormal involuntary movements (ICD-10-CM R25.9, OMOP 45539328 and 376229); Transient alteration of awareness (ICD-10-CM R40.4, OMOP 37160989 and 4275359).

To identify maternal and child conditions concomitant with maternal hypertension and child seizures, respectively, the following codes were used: Maternal Diabetes mellitus (SNOMED CT 73211009, OMOP 436077), Maternal Obesity (SNOMED CT 414915002, OMOP 433736) and Child Developmental delay (SNOMED CT 248290002, OMOP 436077).

Basic descriptive analyses were performed as well as calculation of odds ratios for child seizure for those with and without maternal hypertension. These statistical analyses were performed in Python (version 3.10.14) using the package *statsmodels* (version 0.14.2). A multivariable logistic regression was also performed evaluating the odds of child seizure among mothers with hypertension compared to mothers without hypertension, adjusted for maternal age, BMI, diabetes, and child ancestry. This analysis was performed in R (version 4.4.2).

International validation cohort study

Our international validation, population-based study utilized the National Health Insurance Research Database (NHIRD) of Taiwan, which contains insurance claims data of beneficiaries under the National Health Insurance (NHI) Program. These data cover up to 99.9% of Taiwan's population (2). To enhance and centralize available data, Taiwan's Ministry of Health and Welfare established the Health and Welfare Data Center (HWDC) to link the NHIRD with other health-related databases, including the Maternal and Child Health Database and the Birth Registry Database. Here, we linked maternal-child records from the Maternal and Child Health Database and the NHIRD Birth Registry Database during the period 2000 to 2015. Eligible children were required to have at least two follow-up visits within any two-year interval before reaching 18 years of age to ensure adequate health monitoring. Stillbirths and multiple births

from the same delivery were excluded. This study was approved by the Institutional Review Board of China Medical University Hospital (CMUH111-REC2-155).

The primary exposure of interest was maternal hypertension during pregnancy, which included both pre-existing hypertension and gestational hypertension. Pregnancy duration was determined based on the gestational weeks recorded in the Birth Registry Database. The primary outcome was childhood seizure diagnosis before 18 years of age. Maternal hypertension and childhood seizures were identified using the relevant ICD-9 and ICD-10 diagnosis codes listed in **Supplemental Table 11**. Covariates included maternal age at delivery from the Birth Registry Database, and diabetes mellitus and obesity status, as defined by appropriate ICD-9 and ICD-10 diagnosis codes (**Supplemental Table 11)**. Delivery-related covariates included gravida, gestational weeks, infant's sex, birth weight delivery method (e.g., vaginal delivery or C-section), APGAR scores, congenital defects, delivery complications, and pregnancy procedures obtained from the Birth Registry Database.

For this cohort's descriptive analyses, categorical variables are presented as frequency (proportion) and analyzed using the chi-square test, while the continuous variables are presented as median (first and third quantile) and analyzed using the Wilcoxon rank sum test. The association of maternal hypertension with the risk of childhood seizure was investigated using both a multivariate logistic regression model and a Cox proportional hazards model, adjusting for maternal variables of age, diabetes, obesity, and gestational weeks, and infant variables of birth weight, 5th-minute APGAR Score, and development delay occurred before the seizure event. In the Cox model, a competing risk analysis was performed using a cause-specific Cox proportional hazards model, with death regarded as the censoring event. A two-sided α value of 0.05 indicated statistical significance. All statistical analyses were performed using SAS (version 9.4; SAS

Institute Inc., Cary, NC, USA) in a secured area of the Data Science Center, Ministry of Health and Welfare, Taiwan.

Animal studies

Eighty female and forty male mice (C57Black/6J, 10-week-old, Jackson Lab.) were used for breeding. The dams were instrumented with telemetry probes (TA11PA-C10; Data Sciences International) through the carotid artery for continuous monitoring of blood pressure (BP) and heart rate (HR). After baseline BP and HR recordings were made, female dams were implanted with subcutaneous osmotic minipumps (Alzet, #1004) loaded with vehicle (saline), angiotensin (ANG) II (subcutaneous, 1200 ng/kg/min, 4 weeks), or phenylephrine (PE, subcutaneous, 60 μg/kg/min, 4 weeks), which delivered their contents continuously throughout mating and pregnancy. Offspring were weighed and counted at birth, and both male and female offspring were used in all experiments. Experimental animals were randomly selected and balanced across litters.

At four weeks of age, a subset of male and female offspring from normotensive (saline) or hypertensive (ANG II) dams were euthanized, and the brains were collected for molecular and histologic analyses (n=5-6/group). At 10-12 weeks of age (adult), non-treated offspring from saline, ANG II or PE dams, and offspring from saline or ANG II dams which had received PTX in the drinking water or PLX 5622 in the food, were euthanized and processed in the same way to determine changes in hippocampal inflammation (n=5-6/group).

In separate experiments, at 4-5 weeks (young) or 10-12 weeks (adult) of age, male and female offspring of saline, ANG II and PE dams were used to determine whether maternal hypertension during pregnancy resulted in an increased risk of seizure and to evaluate the effects of anti-

inflammatory treatment or microglial depletion on seizure risk. Young male and female offspring were divided into 4 groups (n=8-11/group/sex): 1) offspring of saline dams treated with the seizure inducer, Pilocarpine (Pilo), 2) offspring of ANG II dams with Pilo, 3) offspring of saline dams with electrical stimulation (ES), 4) offspring of ANG II dams with ES. Sexes were tested separately in all cases. In adult offspring, chemical seizure induction experiments included a total of 7 groups (n=7-9/group/sex): 1) offspring of saline dams with Pilo, 2) offspring of ANG II dams with Pilo, 3) pentoxifylline (PTX)-treated offspring of saline dams with Pilo, 4) PTX offspring of ANG II dams with Pilo, 5) PLX5622 offspring of saline dams with Pilo, 6) PLX5622 offspring of ANG II dams with Pilo, 7) offspring of PE dams with Pilo. Sexes were tested separately in all cases. For experiments using ES, adult male and female offspring were divided into 9 groups (n=7-11/group/sex): 1) offspring of saline dams with ES, 2) offspring of ANG II dams with ES, 3) diazepam-treated offspring of saline dams with ES, 4) diazepam offspring of ANG II dams with ES, 5) PTX offspring of saline dams with ES, 6) PTX offspring of ANG II dams with ES, 7) PLX5622 offspring of saline dams with ES, 8) PLX5622 offspring of ANG II dams with ES, 9) offspring of PE dams with ES. Sexes were tested separately in all cases. Finally, chronic EEG recordings (10 weeks) in adult offspring after chemical seizure induction included a total of 2 groups (n=4/group/sex): 1) offspring of saline dams with Pilo and 2) offspring of ANG II dams with Pilo.

Electrode implantation, radiotelemetry setup and video-EEG recording

At 14-16 weeks of age, male and female offspring of saline and ANG II dams were used to determine whether maternal hypertension during pregnancy resulted in an increased risk of seizure. Offspring were given consecutive injections of pilocarpine (50 mg/ kg, ip) at an interval

of 20 minutes until a total of 6 doses were administered. Seizures were scored behaviorally on a modified Racine scale, as described previously (3).

Two weeks following the induction of chemical seizures, offspring were implanted with ETA-F20 PhysioTelTM radio-transmitters (Data Science International, CAT: 270-0042-001). The detailed procedure for electrode implantation, radiotelemetry setup and recording are described in our previous and recent publications (4).

One day after electrode implantation, mice were moved into the telemetry room and were allowed to acclimate for 7 days before the commencement of recording. Following the acclimation period, mice were continuously recorded for 94 days using Ponemah software, version 6.51. After data acquisition, EEG data was analyzed for seizures.

We developed a novel MATLAB-based algorithm that analyzes spiking activity and seizure events to quantify mouse EEG data. The detailed procedure for our algorithm-based automatic quantification of electrographic spikes and seizures was recently published (5). All seizures were verified against the power spectrum (high-frequency gamma bands), by electromyogram, and against videos synchronized to EEG, as well as via this MATLAB-based algorithm.

Diazepam, PTX or PLX 5622 treatment

Diazepam (5 mg/kg, i.p.) was given to offspring 30 minutes before electrical stimulation. At 8 weeks of age, offspring from saline or ANG II dams were given PTX (1.3 mg/mL, Sigma) in their drinking water for two weeks, which results in a PTX dose of 150 mg/kg/day. At 9 weeks of age, offspring were provided *ad libitum* access to PLX5622 or vehicle diet for 7 days. PLX5622 was formulated in AIN-76A rodent chow by Research Diets (New Brunswick, NJ) at a concentration of 1200 ppm. Standard AIN-76A diet was provided as vehicle control.

Induction of chemical seizures

After an acclimation period in the recording chamber of at least 1 h, animals received scopolamine methyl nitrate (1 mg/kg, ip) to reduce the peripheral effects of pilocarpine. After 15 min, animals received the first dose of pilocarpine (50 mg/ kg, ip). Subsequent pilocarpine injections (50 mg/ kg, ip) were given every 20 min until a cumulative dose of 500 mg/kg was reached.

Seizures were scored behaviourally on a modified Racine scale as follows (3): 0, behavioral arrest; 1 and 2 (grouped together), facial automatisms, tremor, tail stiffening, head bobbing, body jerks; 3, single limb myoclonus; 4, bilateral myoclonus, rearing, non-sustained tonic–clonic activity; 5, recurrent (<2 min apart), sustained tonic–clonic seizures (status epilepticus) and/or extension; 6, death.

Electrical seizure induction

After an acclimation period of 15 min, offspring received electroshock stimulations via ear-clip electrodes connected to a Rodent Shocker 221 (Harvard Apparatus, Holliston MA, USA). The sensitivity and lethality of seizures were determined by stimulating offspring with increasing current (1, 3, 5, 10 mA; 5 min between stimulations) until seizures and/or death occurred. Seizures were scored behaviorally on a modified Racine scale, as described in the “Chemical seizure induction” section.

Quantitative real-time polymerase chain reaction (RT-PCR)

Total RNA was extracted from the hippocampus of saline and ANG II offspring using TRIzol (Invitrogen) per the manufacturer’s instructions. RNA yield and purity were evaluated using a

NanoDrop spectrophotometer. First-strand complementary DNA (cDNA) was synthesized with SuperScript III reverse transcriptase (Invitrogen). Amplified cDNAs were diluted 1:15 in ultra-pure water and subjected to PCR on an Applied Biosystems QuantStudio 7 Flex or Quant Studio 7 Pro with TaqMan Universal PCR Mastermix (Applied Biosystems, Foster City, CA) and the following Taqman probes: *Tnf* (Mm00443258), *Cd11b* (Mm00434455), and *Gapdh* (Mm99999915). PCR reactions were conducted as follows: 2 min at 50°C, 10 min at 95°C, followed by 40 cycles for amplification at 95°C for 15 sec and 60°C for 60 sec. Biological samples were run in duplicate. Genes of interest were normalized to the endogenous control *Gapdh*. The final concentration of mRNA was calculated using the formula $x=2^{\wedge(-\Delta\Delta Ct)}$, and results were expressed as fold difference from saline controls.

Expression of ANG II type 1 receptor (*At1r*) mRNA and of *Gapdh* mRNA was assessed via SYBR Green real time PCR (RT-PCR). In total, 200-300 ng of cDNA and 500 nM of each primer were combined in a 20 µL reaction with iQ SYBR Green Supermix (Bio-Rad). RT-PCR cycles were as follows: 95°C for 3 min, followed by 40 cycles of 95°C for 15 s and annealing/extension at 60°C for 30 s. Reactions were performed in duplicate and analyzed using a C1000 thermocycler system (Bio-Rad). The mRNA values for *At1r* were normalized to *Gapdh* and the final concentration of mRNA was calculated using the formula $x=2^{\wedge(-\Delta\Delta Ct)}$, where x=fold difference relative to controls. Primers were purchased from Integrated DNA Technologies (Coralville, IA). Primer sequences were: *At1r* (forward, TGCCCATAACCATCTGCATAG, reverse, TTTCAGGAGCTGGAGGAAATAC); *Gapdh* (forward, AGGTTGTCTCCTGCGACTTCA, reverse, CCAGGAAATGAGCTTGACAAAG)

Fluorescent immunohistochemistry

Coronal brain sections (10 µm) were incubated with a rabbit macrophage/microglia-specific polyclonal anti-Iba-1 antibody (1:400, Cat. GTX 10042, GeneTex) in 10% donkey normal serum with 0.2% Triton X-100 overnight at 4°C. After being thoroughly washed with PBS, sections were incubated with donkey anti-rabbit Alexa Flour 594 (1:400; Cat. A-21207, Thermo Fisher) in PBS for 1 h. Sections were then mounted and counterstained with DAPI. In each animal, cells that were positive for both the nuclear DAPI and Iba-1 were counted manually, and the counts of two representative 10-µm transverse sections at the level of the hippocampus were averaged.

Data Analysis

For analysis of human data from the Epic Cosmos dataset, the Chi-square contingency test with Yates correction was employed. Odds ratios (OR) and corresponding 95% confidence intervals (CI) were calculated.

In animal studies, mean arterial pressure (MAP), HR, and locomotor activity are presented as mean daily values. Two-way ANOVAs by condition were conducted on daily MAP, HR or activity, and significant ANOVAs were followed by post-hoc Tukey's multiple comparison tests. The same statistical approach was used for Racine scale results. Offspring survival after application of pilocarpine or electrical stimulation was analyzed by the Log-rank (Mantel-Cox) test. One-way ANOVAs followed post-hoc Tukey multiple comparison tests or Kruskal-Wallis tests followed by Dunn's multiple comparison tests (non-normally distributed data) were used to compare differences in litter size, pup birthweight, and gene expression between groups. All data were expressed as means ± SE. Statistical significance was set at $p < 0.05$. Outliers were tested using the "identify outliers" function in GraphPad Prism 10.4.1 and were removed.

References

1. Reinecke I, Zoch M, Reich C, Sedlmayr M, and Bathelt F. The Usage of OHDSI OMOP - A Scoping Review. *Stud Health Technol Inform.* 2021;283:95-103.
2. Hsieh CY, Su CC, Shao SC, Sung SF, Lin SJ, Kao Yang YH, et al. Taiwan's National Health Insurance Research Database: past and future. *Clin Epidemiol.* 2019;11:349-58.
3. Buchanan GF, Murray NM, Hajek MA, and Richerson GB. Serotonin neurones have anti-convulsant effects and reduce seizure-induced mortality. *J Physiol.* 2014;592(19):4395-410.
4. Rodriguez S, Sharma S, Tiarks G, Peterson Z, Jackson K, Thedens D, et al. Neuroprotective effects of naltrexone in a mouse model of post-traumatic seizures. *Sci Rep.* 2024;14(1):13507.
5. Kyle JJ, Sharma S, Tiarks G, Rodriguez S, and Bassuk AG. Fast Detection and Quantification of Interictal Spikes and Seizures in a Rodent Model of Epilepsy Using an Automated Algorithm. *Bio Protoc.* 2023;13(6):e4632.

Supplemental Table 1. Characteristics of mothers and children in the Epic Cosmos patient database from January 1, 2011, to December 31, 2023.

**Birthing Mother's Diagnosis during pregnancy**	**Measures**<br><br>**(SNOMED codes)**	**Seizures in Child, ICD-10-CM codes: G40, R56, P90, R25.9, R40.4**	**No Seizures in Child**	**Total**
**Hypertension during pregnancy**<br><br>**ICD-10-CM codes: O14, I10, O11, O13, O16, I15, O10.**	n	50,288	1,314,966	1,365,254
	Female	44.6%	48.5%	48.4%
	Maternal Obesity (414915002)	43%	40.5%	40.6%
	Advanced Maternal Age	19.4%	21.6%	21.6%
	Maternal Diabetes (73211009)	20.4%	18.5%	18.6%
	Developmental Delay (248290002)	31.3%	9.5%	10.3%
**No Hypertension during Pregnancy**	n	179,069	5,712,755	5,891,824
	Female	45%	48.8%	48.6%
	Maternal Obesity (414915002)	16.3%	15.3%	15.3%
	Advanced Maternal Age	14.3%	16.9%	16.8%
	Maternal Diabetes (73211009)	8.4%	8.2%	8.2%
	Developmental Delay (248290002)	25.8%	7.1%	7.7%
**Total**	n	229,357	7,027,721	7,257,078
	Female	44.9%	48.7%	48.6%
	Maternal Obesity	22.2%	20%	20.1%
	Advanced Maternal Age	15.5%	17.8%	17.7%
	Maternal Diabetes	11%	10.2%	10.2%
	Developmental Delay	27%	7.6%	8.2%

Supplemental Table 2. Maternal age at delivery in the Epic Cosmos patient database from January 1, 2011, to December 31, 2023.

Diagnosis	Mothers with seizure children		Mothers without seizure children		Total	
**Measures**	Average Maternal Age	Count	Average Maternal Age	Count	Average Maternal Age	Count
**Maternal Hypertension**	29 years	54,257	30 years	1,428,983	30 years	1,483,240
**No Maternal Hypertension**	28 years	177,326	29 years	5,740,185	29 years	5,917,511
**Total**	28 years	231,583	29 years	7,169,168	29 years	7,400,751

Supplemental Table 3. Maternal ancestry in the Epic Cosmos patient database from January 1, 2011, to December 31, 2023 (patients can self-report in more than 1 ancestry/ethnicity category)

Maternal Ancestry	Mothers with hypertension during pregnancy	Mothers without hypertension during pregnancy	Total
**Non-Hispanic White**	756,125	3,205,400	3,961,525
**Non-Hispanic AA/Black**	328,917	967,270	1,296,187
**Hispanic**	193,682	1,022,750	1,216,432
**Asian**	46,283	355,778	402,061
**Other Ancestry**	124,193	762,794	886,987
**Total**	1,394,863	6,005,888	7,400,751

Supplemental Table 4. Child ancestry in the Epic Cosmos patient database from January 1, 2011, to December 31, 2023 (patients can self-report in more than 1 ancestry/ethnicity category)

Child Ancestry	Children from maternal hypertensive mothers	Children from normotensive mothers	Total
**Non-Hispanic White**	734,922	3,187,003	3,921,925
**Non-Hispanic AA/Black**	336,226	1,025,607	1,361,833
**Hispanic**	196,360	1,008,628	1,204,988
**Asian**	41,567	323,696	365,263
**Other Ancestry**	203,737	1,036,275	1,240,012
**Total**	1,394,863	6,005,888	7,400,751

Supplemental Table 5. Iowa Intergenerational Health Knowledgebase Cohort Characteristics

	Control<br>N=34297	Neonatal Seizure<br>N=1370	Test Statistic
**Average Maternal Age**	29.8 ± 8.0	29.2 ± 5.8	P=0.006
**Gravida**	2.8 ± 1.9	2.9 ± 2.2	P=0.052
**BMI**	31.2 ± 0.33	31.5 ± 0.23	P=0.897
**BMI > 30**	27.2%	25.0	P=0.074
**BMI > 40**	12.3%	15.9%	P<0.001
**Minority**	27.2%	25.0%	P=0.074
**ANO**	35.1%	58.7%	P<0.001
**CHTN**	14.1%	16.9%	P=0.005
**Preeclampsia**	11.6%	13.6%	P=0.030
**SIPE**	2.3%	3.2%	P=0.037
**GHTN**	14.7%	15.7%	P=0.038
**Preexisting HTN**	9.9%	12.0%	P=0.013
**HDP**	32.9%	37.2%	P<0.001
**All Diabetes**	15.5%	17.0%	P=0.342
**Birthweight (g)**	3142 ± 806	3167 ± 819	P=0.257
**APGAR 1**	7.3 ± 2.0	6.4 ± 2.6	P<0.001
**APGAR 5**	8.5 ± 1.5	8.0 ± 1.7	P<0.001
**Gestational Age at Delivery (days)**	267 ± 179	264 ± 221	P=0.571

BMI, Body Mass Index; ANO, Adverse Neonatal Outcomes; CHTN, Chronic Hypertension; SIPE, Superimposed Preeclampsia; GHTN, Gestational Hypertension; HTN, Hypertension; HDP, Hypertensive Disorders of Pregnancy.

Supplemental Table 6. Hypertensive diseases of pregnancy are associated with a higher risk of seizures in offspring after controlling for some significant covariates in the Iowa Intergenerational Health Knowledgebase study.

Dependent Variable: Offspring Seizure	aOR	95% CI	Test Statistic
**Independent Variables**			
**Maternal Age**	0.987	(0.0423-0.0618)	P<0.001
**Gravida**	1.037	(1.007-1.067)	P=0.015
**BMI > 40**	1.241	(1.057-1.457)	P=0.008
**Hypertensive Disease in Pregnancy**	1.132	(1.003-1.278)	P=0.045
**Diabetes in Pregnancy**	1.111	(0.955-1.293)	P=0.171
**Minority**	0.877	(0.770-0.998)	P=0.047

BMI, Body Mass Index; ANO, Adverse Neonatal Outcomes; CHTN, Chronic Hypertension; SIPE, Superimposed Preeclampsia; GHTN, Gestational Hypertension; HTN, Hypertension; HDP, Hypertensive Disorders of Pregnancy.

Supplemental Table 7. Stanford validation study regression. Maternal hypertension is a risk factor for childhood seizure, even after controlling for additional covariates by logistic regression. Offspring seizure is the dependent variable, independent variables are listed below.

	aOR (for offspring seizure)	95% CI	Test Statistic
**Independent Variables:**			
**Maternal Age**	0.985	(0.977, 0.993)	$p<0.001$
**BMI > 40**	1	(0.994, 1.001)	$p=0.99$
**Diabetes in Pregnancy**	1.209	(1.073, 1.358)	$p=0.002$
**Non-Hispanic Asian**	0.906	(0.798, 1.029)	$p=0.129$
**Non-Hispanic Black**	1.435	(1.076, 1.881)	$p=0.011$
**Hispanic**	1.235	(1.093, 1.397)	$p<0.001$
**Other ancestry**	0.674	(0.560, 0.807)	$p<0.001$
**Hypertensive Disease in Pregnancy**	1.358	(1.206, 1.524)	$p<0.001$

Supplemental Table 8. Stanford validation study cohort characteristics.

		Seizures in Child[#]	No Seizures in Child	Total
**Hypertension during pregnancy***	*n*	399	7,620	8,019
	Female	43.60%	48.10%	47.87%
	Maternal Obesity	30%	22.85%	23.12%
	Advanced Maternal Age	36.84%	38.23%	38.16%
	Maternal Diabetes	31.10%	28.00%	28.16%
	Developmental Delay	0.00%	0.00%	0.00%
**No Hypertension during Pregnancy**	*n*	1,678	45,927	47,605
	Female	43%	48.29%	48.10%
	Maternal Obesity	11.38%	6.90%	7.05%
	Advanced Maternal Age	30.75%	34.54%	34.40%
	Maternal Diabetes	16.27%	13.34%	14.41%
	Developmental Delay	0.42%	0.00%	0.03%
**Total**	n	2,077	53,547	55,624
	Female	42.85%	48.26%	48.06%
	Maternal Obesity	14.88%	9.17%	9.38%
	Advanced Maternal Age	31.92%	35.06%	34.94%
	Maternal Diabetes	19.11%	16.29%	16.39%
	Developmental Delay	0.39%	0.01%	0.03%

**ICD-10-CM codes: O14, I10, O11, O13, O16, I15, O10*
#ICD-10-CM codes: G40, R56, P90, R25.9, R40.4

Supplemental Table 9. Taiwanese validation study regression. Maternal hypertension is a risk factor for childhood seizure, even after controlling for additional covariates by logistic regression.

Maternal hypertension	No. of childhood seizure	Incidence (per 10,000 p-y)	Odds ratio (95% CI)	
			Crude	Adjusted*
**No**	104811 (5.50%)	9.08	Ref.	Ref.
**Yes**	6582 (6.85%)	11.33	1.26 (1.23-1.30)	1.17 (1.14-1.20)

*Adjusted for: maternal age, maternal diabetes, maternal obesity, gestational age at delivery, birth weight, infant 5-minute APGAR score, and child's development delay

Supplemental Table 10. Taiwanese validation study cohort characteristics.

Variable	Total N=2003354	Maternal Hypertension N=96142	No Maternal Hypertension N=1907212	P-value
**Maternal**				
Average Age at delivery	30 (27, 33)	31 (27, 34)	30 (27, 33)	<0.0001
Taiwan nationality	1878614 (93.8)	94042 (97.8)	1784572 (93.6)	<0.0001
Diabetes	381367 (19.0)	29558 (30.7)	351809 (18.5)	<0.0001
Obesity	26500 (1.32)	4486 (4.67)	22014 (1.15)	<0.0001
**Birth-related**				
Gestational weeks	39 (38, 39)	38 (37, 39)	39 (38, 39)	<0.0001
Boy	1044519 (52.1)	48610 (50.6)	995909 (52.2)	<0.0001
Birth weight	3100 (2850, 3360)	3000 (2650, 3322)	3100 (2860, 3362)	<0.0001
Delivery by C-section	684234 (34.2)	50961 (53.0)	633273 (33.2)	<0.0001
1st minute APGAR <7	36772 (1.84)	5571 (5.79)	31201 (1.64)	<0.0001
5th minute APGAR <7	5720 (0.29)	1074 (1.12)	4646 (0.24)	<0.0001
Congenital defects				
Neurological	310 (0.02)	16 (0.02)	294 (0.02)	0.765
Ophthalmological and facial	2459 (0.12)	161 (0.17)	2298 (0.12)	<0.0001
Cardiovascular	1162 (0.06)	107 (0.11)	1055 (0.06)	<0.0001
Digestive	900 (0.04)	68 (0.07)	832 (0.04)	0.0001
Urogenital or genitourinary	1108 (0.06)	103 (0.11)	1005 (0.05)	<0.0001
Musculoskeletal	2733 (0.14)	165 (0.17)	2568 (0.13)	0.002
Respiratory	217 (0.01)	17 (0.02)	200 (0.01)	0.037
Chromosomal abnormality	217 (0.01)	17 (0.02)	200 (0.01)	0.037
Others	573 (0.03)	44 (0.05)	529 (0.03)	0.001
**Delivery-related**				

Special procedure during delivery				
Amniocentesis	62627 (3.13)	4105 (4.27)	58522 (3.07)	<0.0001
Chorionic villus sampling	589 (0.03)	43 (0.04)	546 (0.03)	0.005
Labor induction	8991 (0.45)	995 (1.03)	7996 (0.42)	<0.0001
Augmentation	134107 (6.69)	8083 (8.41)	126024 (6.61)	<0.0001
Tocolysis	25838 (1.29)	3433 (3.57)	22405 (1.17)	<0.0001
Cervical cerclage	1835 (0.09)	146 (0.15)	1689 (0.09)	<0.0001
Exploratory laparotomy	1102 (0.06)	64 (0.07)	1038 (0.05)	0.117
Delivery complications				
Fever (> 38ºC)	6742 (0.34)	375 (0.39)	6367 (0.33)	0.003
Moderate or severe meconium	27398 (1.37)	1791 (1.86)	25607 (1.34)	<0.0001
Premature rupture of membrane (>12 hr)	31266 (1.56)	1788 (1.86)	29478 (1.55)	<0.0001
Placental abruption	6492 (0.32)	852 (0.89)	5640 (0.30)	<0.0001
Placenta previa	15546 (0.78)	741 (0.77)	14805 (0.78)	0.849
Massive bleeding	6873 (0.34)	646 (0.67)	6227 (0.33)	<0.0001
Emergency delivery (<3 hr)	37579 (1.88)	1402 (1.46)	36177 (1.90)	<0.0001
Prolonged delivery (>20 hr)	30797 (1.54)	1929 (2.01)	28868 (1.51)	<0.0001
Breech position	81972 (4.09)	4771 (4.96)	77201 (4.05)	<0.0001
Cephalopelvic disproportion	20647 (1.03)	1162 (1.21)	19485 (1.02)	<0.0001
Prolapse of umbilical cord	2145 (0.11)	126 (0.13)	2019 (0.11)	0.020
Fetal distress	23413 (1.17)	3011 (3.13)	20402 (1.07)	<0.0001
**Outcome in children before 18 years old**				
Seizure-related	111393 (5.56)	6582 (6.85)	104811 (5.50)	<0.0001
Development delay	362378 (18.1)	20297 (21.1)	342081 (17.9)	<0.0001
Death	5511 (0.28)	403 (0.42)	5108 (0.27)	<0.0001

Supplemental Table 11. ICD-9 and ICD-10 diagnostic codes for maternal hypertension, childhood seizure disorders, and maternal characteristics.

Diagnosis	ICD-9 codes	ICD-10 codes
**Maternal Hypertension**		
Pre-existing hypertension complicating pregnancy, childbirth, and the puerperium	642.00 – 642.04 (642.0)<br>642.10 – 642.14 (642.1)<br>642.20 – 642.24 (642.2)	O10
Pre-existing hypertension with pre-eclampsia	642.70, 642.71, 642.73	O11
Gestational (pregnancy-induced) hypertension without significant proteinuria	642.3x	O13
Pre-eclampsia	642.40 – 642.43<br>642.50 – 642.52	O14
Unspecified maternal hypertension	642.31, 642.33<br>642.9x	O16
Essential (primary) hypertension	401.0, 401.1, 401.9	I10
Secondary hypertension	405.01, 405.09, 405.11, 405.19, 405.91, 405.99	I15
**Childhood Seizure**		
Epilepsy and recurrent seizures	345.00, 345.01<br>345.10, 345.11<br>345.2<br>345.3	G40

	345.40, 345.41	
	345.50, 345.51	
	345.60, 345.61	
	345.70, 345.71	
	345.80, 345.81	
	345.90, 345.91	
Convulsions, not elsewhere classified	780.31, 780.39	R56
Convulsions of newborn	779.0	P90
Unspecified abnormal involuntary movements	781.0	R25.9
Transient alteration of awareness	780.02	R40.4

Supplemental Figure 1. Maternal ANG II- or PE-induced gestational hypertension. Changes in systolic blood pressure (SBP, **A**), diastolic BP (DBP, **B**) and activity (**C**) of mouse dams after chronic infusion of saline, angiotensin (ANG) II, or phenylephrine (PE) during pregnancy. (Two-way ANOVAs, * $p<0.05$ vs. saline dams; # $p<0.05$ vs. PE dams).

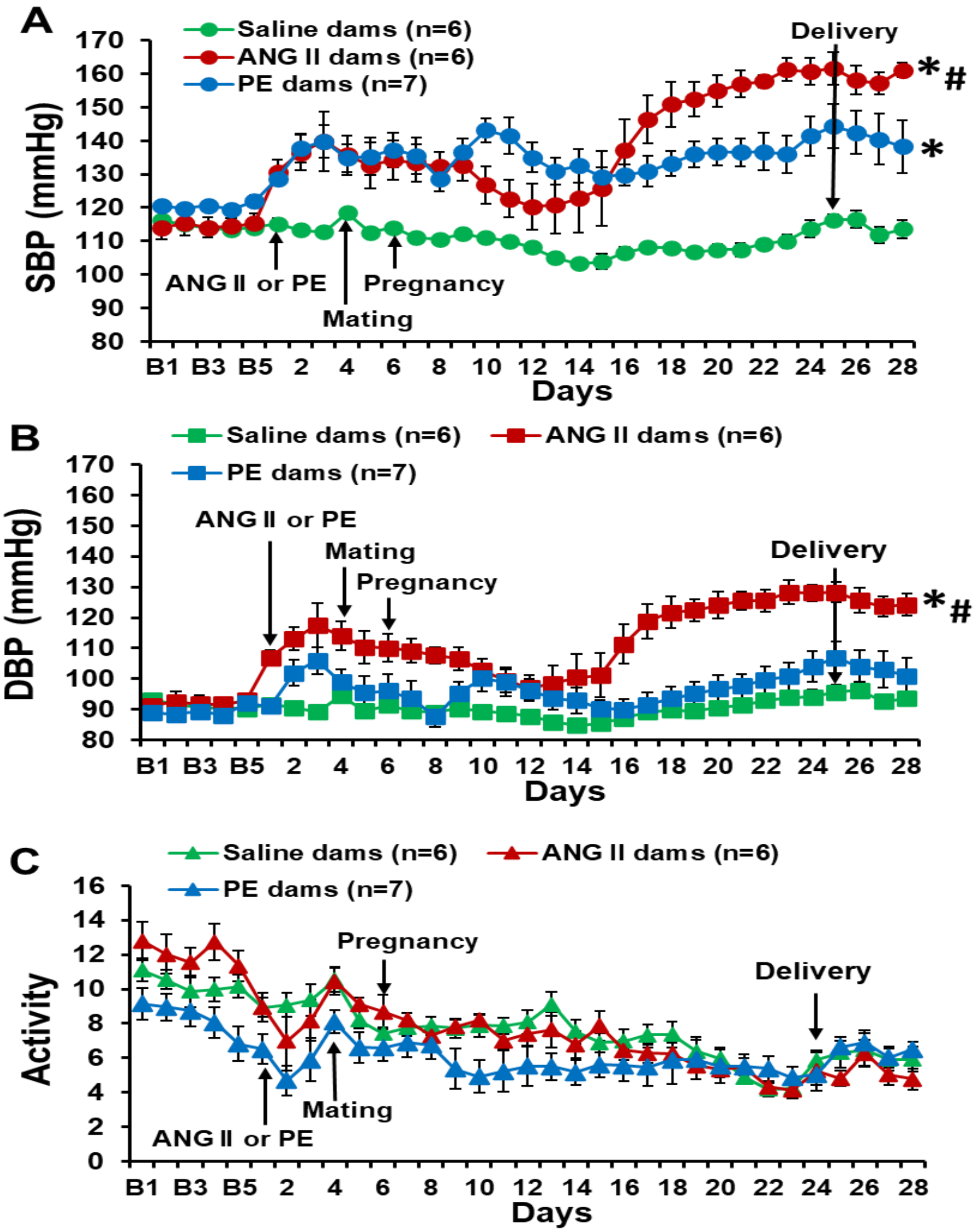

Supplemental Figure 2. Comparison of proinflammatory cytokine (*Tnf*) and a microglial marker (*Cd11b*) mRNA expression in hippocampi of young male and female offspring (4 weeks old, **A**) from normotensive (saline) and maternal angiotensin (ANG) II-induced hypertensive dams (n=5-6/group, Kruskal-Wallis test followed by Dunn's multiple comparisons test). Gestational hypertension increased sensitivity and mortality to pilocarpine-induced (**B**) or electrical stimulation-induced (**C** and **D**) seizures in young offspring (5-6 weeks old, n=8-11, Two-way ANOVAs followed by the Tukey test) * P<0.05 vs. male offspring of saline dams, # p<0.05 vs. female offspring of saline dams, † p<0.05 vs. female offspring of ANG II dams)

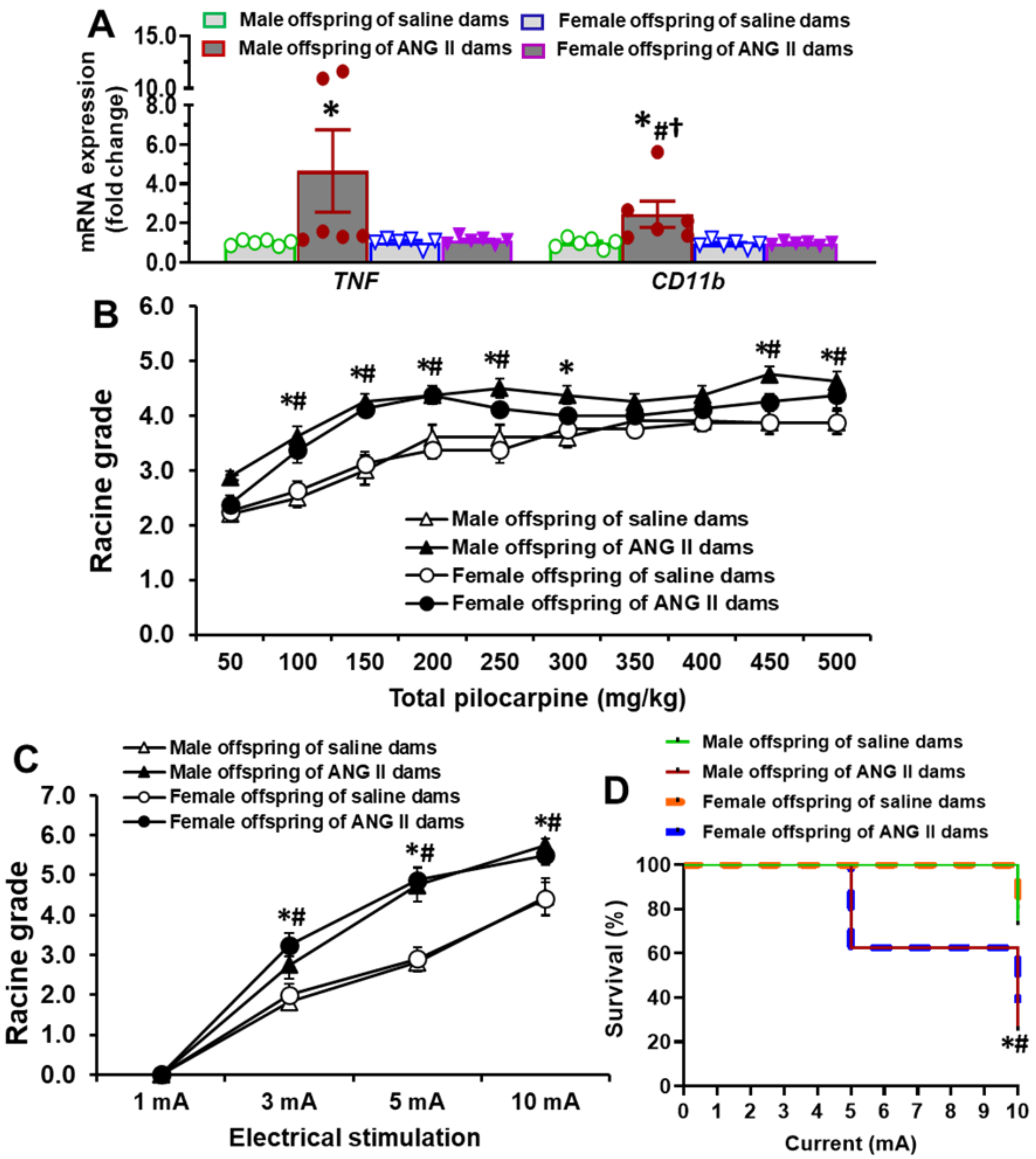

Supplemental Figure 3. Expression of angiotensin II type 1 receptor (*At1r*) mRNA in the hippocampi of male and female offspring from normotensive (saline), maternal angiotensin (ANG) II, or phenylephrine (PE)-induced hypertensive dams (n=5-6/group, One-way ANOVAs followed by Tukey test, ¥ $p<0.05$ vs. other group of offspring)

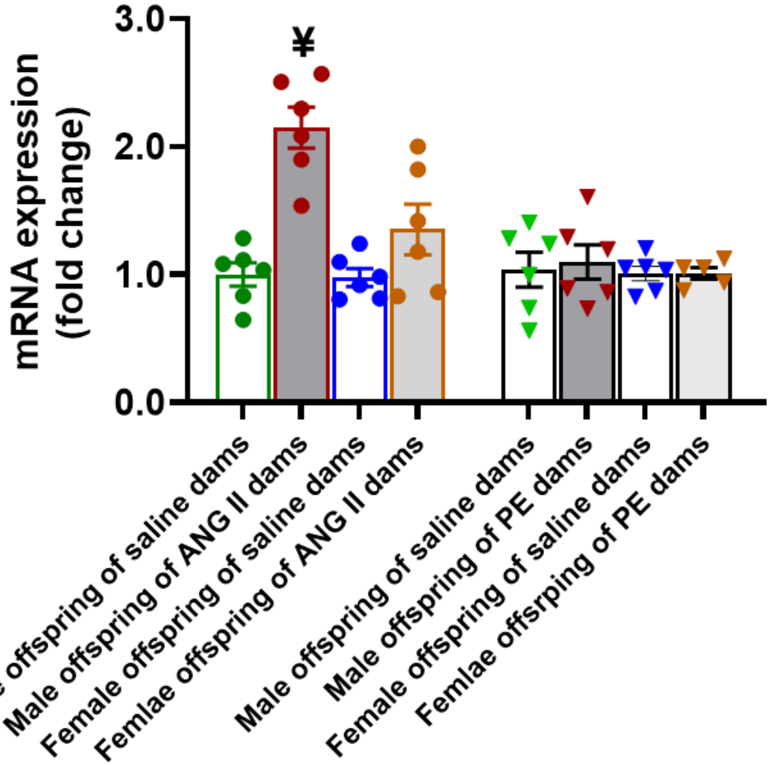

Supplemental Figure 4. Pretreatment with diazepam abolished mortality and significantly attenuated epileptic responses to electrical stimulation in adult male (10 weeks old, **A** and **B**) or female (**C** and **D**) offspring of maternal normotensive (saline) and ANG II-induced hypertensive dams. (n=7-10/group, Two-way ANOVAs followed by the Tukey test, * p<0.05 vs. male or female offspring of saline dams, # p<0.05 vs. diazepam-treated offspring)

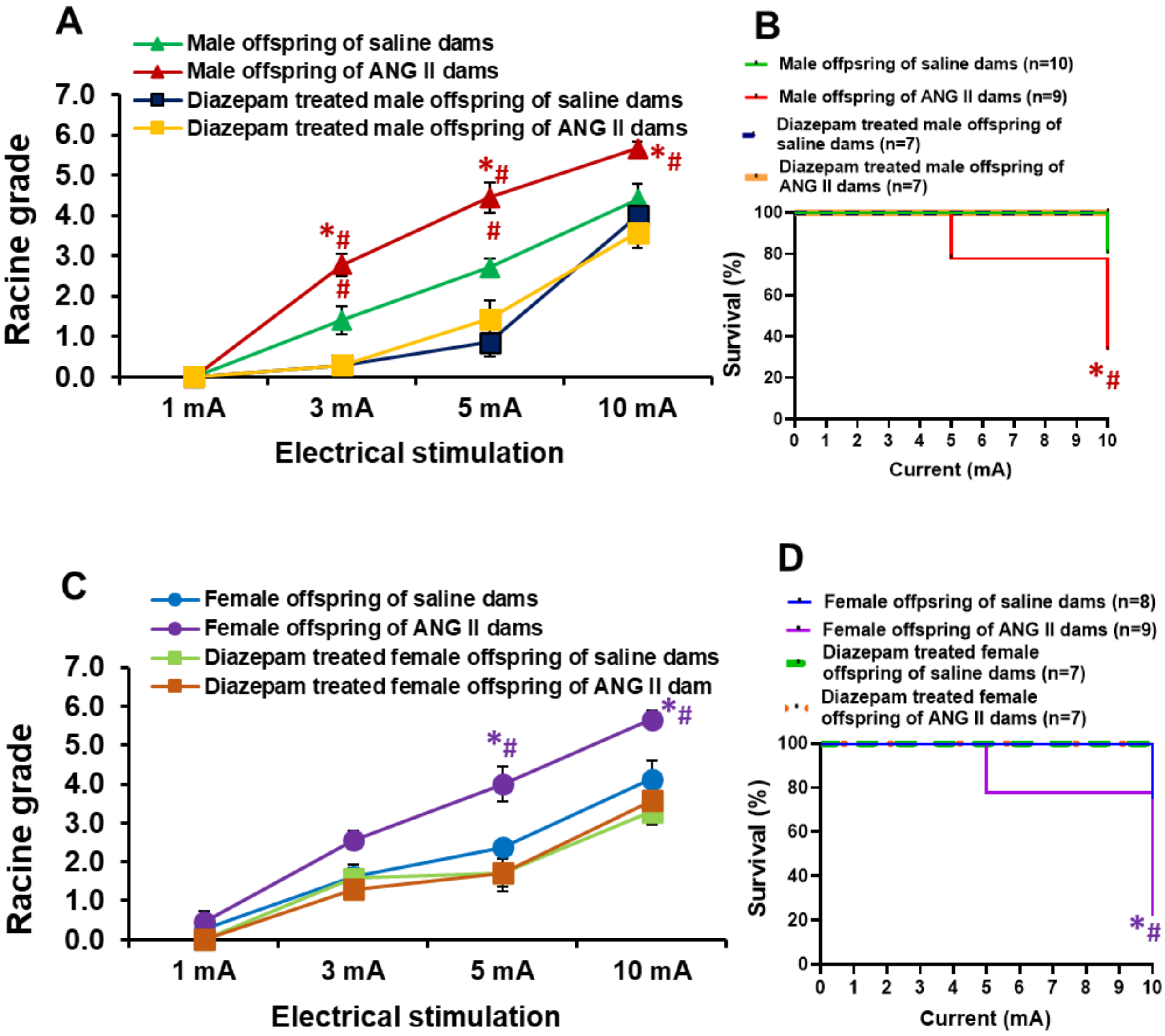

Supplemental Figure 5. Pentoxifylline (PTX) dosing via drinking water for two weeks (**A**). PLX5622 dosing via chow for one week (**C**). Changes in body weight (**B** and **D**) of adult male and female offspring of both normotensive (saline) and gestational hypertensive (ANG II) dams. There were no significant differences among groups (10 weeks old, n=17-20/group, One-way ANOVAs, p>0.05). Linear regression indicating no correlations between PTX (**E**) or PLX5622 (**F**) intake and mRNA expression of *Tnf* and *Cd11b* in the hippocampus.

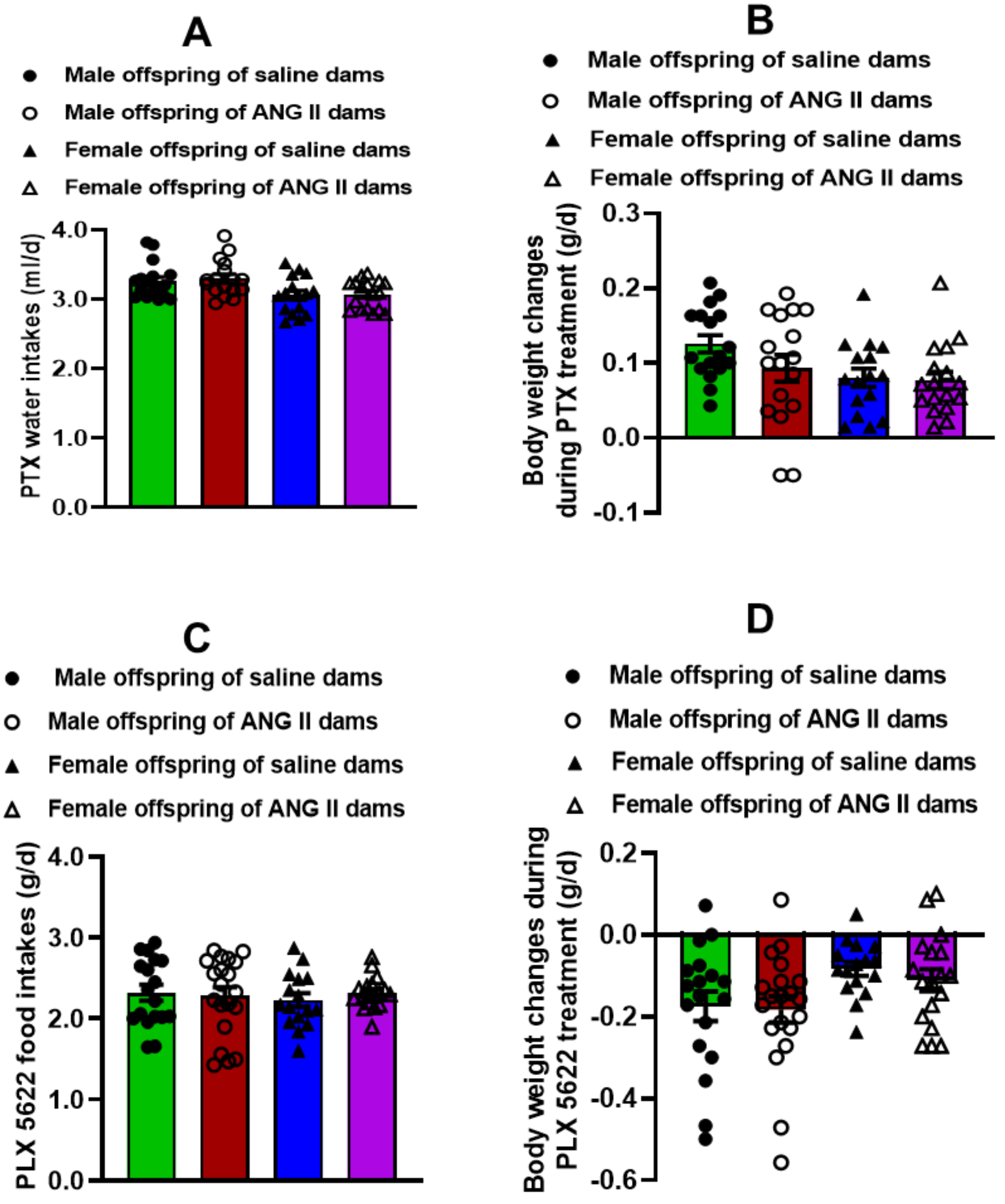

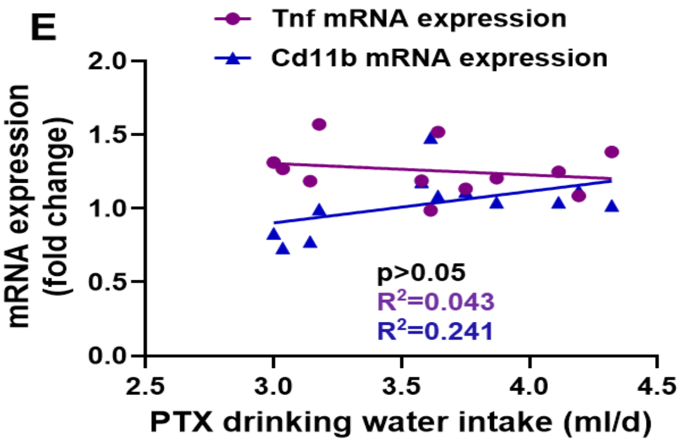
E
Tnf mRNA expression
Cd11b mRNA expression
mRNA expression (fold change)
2.0
1.5
1.0
0.5
0.0
p>0.05
R2=0.043
R2=0.241
2.5
3.0
3.5
4.0
4.5
PTX drinking water intake (ml/d)

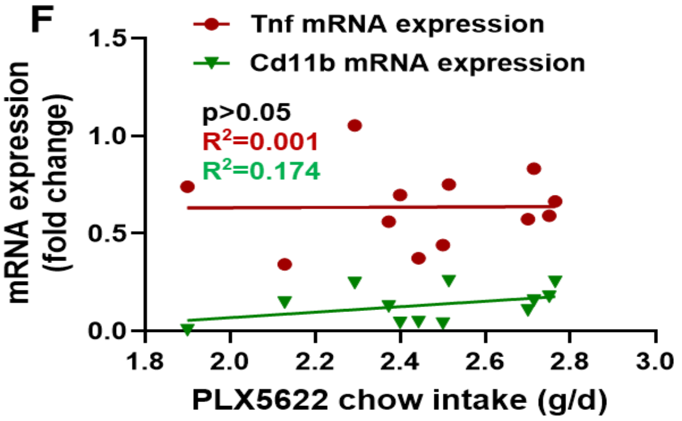
F
Tnf mRNA expression
Cd11b mRNA expression
mRNA expression (fold change)
1.5
1.0
0.5
0.0
p>0.05
R2=0.001
R2=0.174
1.8
2.0
2.2
2.4
2.6
2.8
3.0
PLX5622 chow intake (g/d)

Supplemental Figure 6. Representative immunofluorescent images of microglia (**A**, Iba-1, red) in the hippocampi of adult offspring of normotensive (saline) or hypertensive (ANG II) dams. Cell counting of double labelling of nucleus (blue) and Iba-1 expression (red) demonstrated that oral administration of PLX5622 (1200 ppm) in food for 7 d leads to a significant depletion of microglia across groups (**B**, n=4/group, One-way ANOVAs, * *p*<0.05 vs. control offspring of saline or ANG II dams). Microglia depletion was greater among saline control offspring than among ANG II offspring (**C**, 88.4±2.3% vs. 69.4±3.9%, n=4/group, Mann-Whitney test, # *p*<0.05 vs. PLX5622-treated offspring of saline dams).

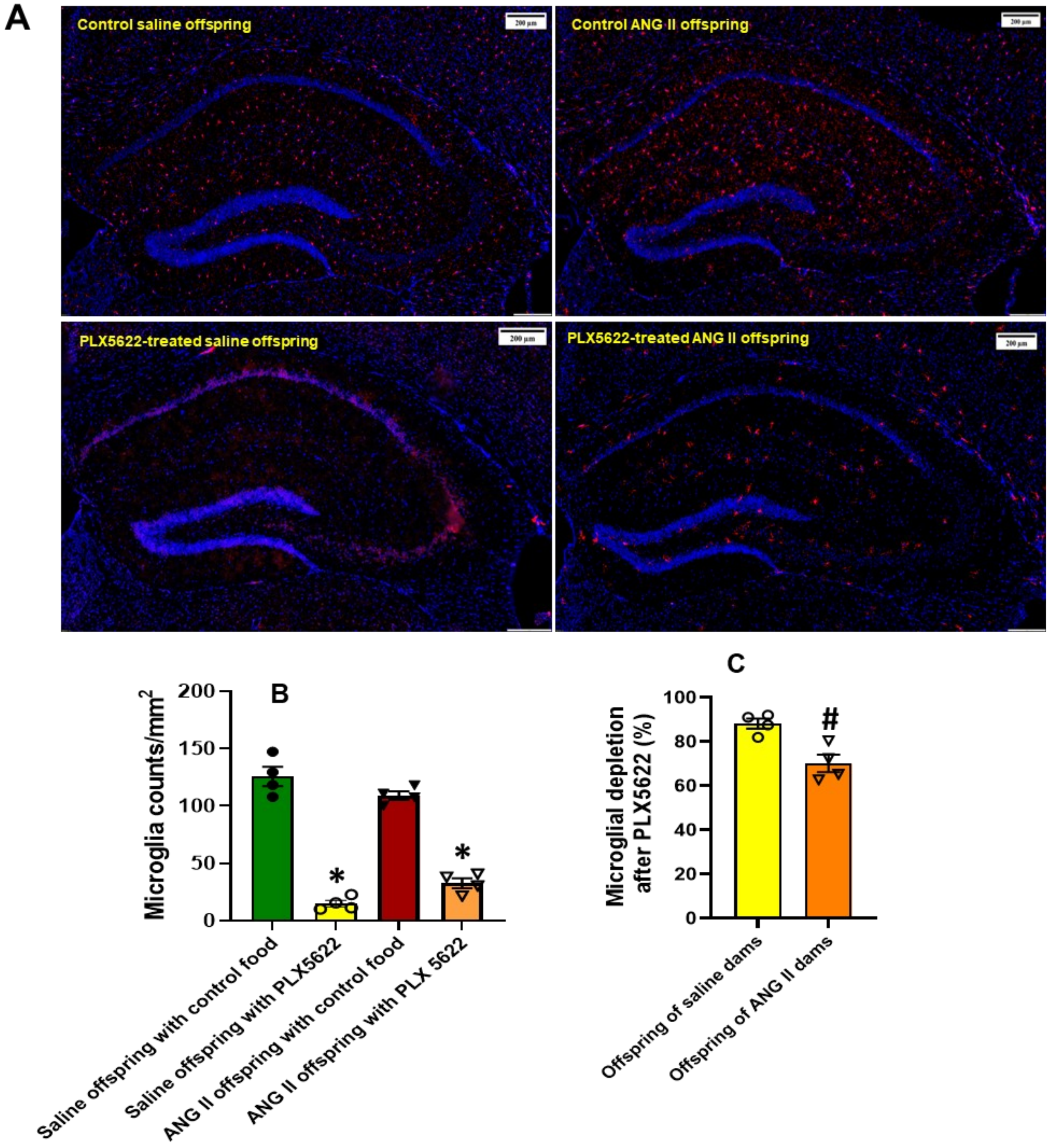

Supplemental Figure 7. Quantitative comparison of seizure frequency at week 10 after pilocarpine injection in male and female offspring from normotensive (saline) and maternal angiotensin (ANG) II-induced hypertensive dams (n=7-8/group including 3-4 males and females per group, Mann-Whitney test, *p<0.05 vs. saline offspring).

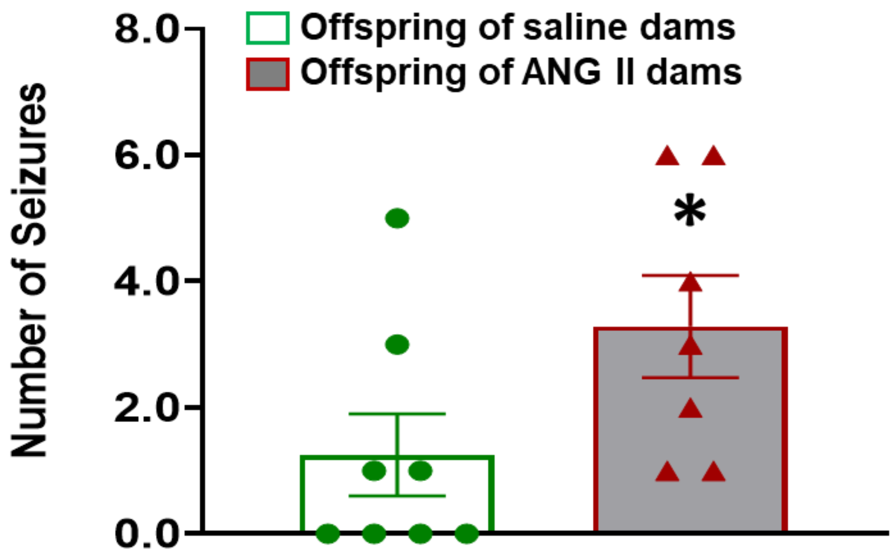

Supplement: Supplemental data [file jci-135-183393-s075.pdf]
